# Infrared: a declarative tree decomposition-powered framework for bioinformatics

**DOI:** 10.1186/s13015-024-00258-2

**Published:** 2024-03-16

**Authors:** Hua-Ting Yao, Bertrand Marchand, Sarah J. Berkemer, Yann Ponty, Sebastian Will

**Affiliations:** 1 LIX, CNRS UMR 7161, Ecole Polytechnique, Institut Polytechnique de Paris, Palaiseau, France; 2 Department of Theoretical Chemistry, University of Vienna, Vienna, Austria; 3 School of Computer Science, McGill University, Montreal, Canada; 4 Earth-Life Science Institute, Tokyo Institute of Technology, Tokyo, Japan

**Keywords:** Bioinformatics, Fixed-parameter tractable algorithms, Tree decomposition, Boltzmann sampling, Network phylogeny, RNA sequence design, RNA alignment, Pseudoknots

## Abstract

**Motivation:**

Many bioinformatics problems can be approached as optimization or controlled sampling tasks, and solved exactly and efficiently using Dynamic Programming (DP). However, such exact methods are typically tailored towards specific settings, complex to develop, and hard to implement and adapt to problem variations.

**Methods:**

We introduce the Infrared framework to overcome such hindrances for a large class of problems. Its underlying paradigm is tailored toward problems that can be declaratively formalized as sparse feature networks, a generalization of constraint networks. Classic Boolean constraints specify a search space, consisting of putative solutions whose evaluation is performed through a combination of features. Problems are then solved using generic cluster tree elimination algorithms over a tree decomposition of the feature network. Their overall complexities are linear on the number of variables, and only exponential in the treewidth of the feature network. For sparse feature networks, associated with low to moderate treewidths, these algorithms allow to find optimal solutions, or generate controlled samples, with practical empirical efficiency.

**Results:**

Implementing these methods, the Infrared software allows Python programmers to rapidly develop exact optimization and sampling applications based on a tree decomposition-based efficient processing. Instead of directly coding specialized algorithms, problems are declaratively modeled as sets of variables over finite domains, whose dependencies are captured by constraints and functions. Such models are then automatically solved by generic DP algorithms. To illustrate the applicability of Infrared in bioinformatics and guide new users, we model and discuss variants of bioinformatics applications. We provide reimplementations and extensions of methods for RNA design, RNA sequence-structure alignment, parsimony-driven inference of ancestral traits in phylogenetic trees/networks, and design of coding sequences. Moreover, we demonstrate multidimensional Boltzmann sampling. These applications of the framework—together with our novel results—underline the practical relevance of Infrared. Remarkably, the achieved complexities are typically equivalent to the ones of specialized algorithms and implementations.

**Availability:**

Infrared is available at https://amibio.gitlabpages.inria.fr/Infrared with extensive documentation, including various usage examples and API reference; it can be installed using Conda or from source.

**Supplementary Information:**

The online version contains supplementary material available at 10.1186/s13015-024-00258-2.

## Background

Typical applications of computational, “bioinformatical” methods to real world biological problems have inherently high complexity at different levels. For example, these include the design of functional control elements for biotechnology [1, 2], identifying homologies in the context of RNA pseudoknots [3, 4], or the prediction of phylogenies considering complex inheritance patterns [5]. First, *modeling complexity* is directly inherited from the complexity of the biological backdrop. This requires bioinformatics approaches to deal with hard constraints and soft requirements. Moreover, many approaches need to target complex scores, often composed of multiple interdependent objectives, e.g. for predicting optimal solutions or generating designs. In turn, the high modeling complexity is reflected in coding challenges and leads to high *computational complexity* of exact solutions. Frequently, this turns bioinformaticians away from exact, combinatorial methods to less controlled heuristics, for example optimization by local search or genetic algorithms [6], or sampling by MCMC (Markov Chain Monte Carlo)-like approaches [7, 8], which sacrifice guarantees on the optimality of solutions or the time and space complexity of the computations.

Here, we introduce the framework Infrared to cope with these levels of complexity. This system lets users specify a large class of computational problems and solves them by combinatorial algorithms with parameterized complexity [9]. These methods guarantee exactness and work efficiently, when the “complexity” (treewidth) of the problem instance is fixed. In practice, this limits the system to problems with moderately sparse dependencies. The system combines various concepts of computer science, comprising constraint modeling [10], constraint and scoring networks [11, 12], tree decomposition [13], parameterized complexity [9, 14], random generation, and (multidimensional) Boltzmann sampling [15]. Along with Infrared, we advocate and hope to promote the use of exact methods. In place of heuristic methods, many NP-hard problems can be solved by algorithms of parameterized complexity, which our system makes more accessible due to proper abstractions. Other problems profit from building heuristic methods on top of exact algorithms.

Historically, the presented framework originated as a generalization of our own previous work on multitarget RNA design (RNARedprint [16]) and our original approach has been completely reimplemented and extended based on Infrared (RNARedprint v2^1^). In parallel to the presented research, we used an early version of the system for original research in the area of negative RNA design (RNAPond, [17]). Other recent work has strong conceptual ties: Tree-Diet [18] (by using RNAPond and LicoRNA [3]) and AutoDP [19]). Moreover, as we show in this text, sequence and RNA sequence-structure alignment can be implemented following the models of LicoRNA [3] and [20]; both papers introduced closely related solving strategies for alignment.

Our framework aims to facilitate the implementation of complex algorithms based on the declarative modeling paradigm. Instead of implementing a concrete algorithm, it allows users to formally describe the problem by specifying the admissible solutions and their quality assessments. Similar to, e.g. constraint programming or integer linear programming systems, those models are solved automatically by a built-in general mechanism.

**Fig. 1:**
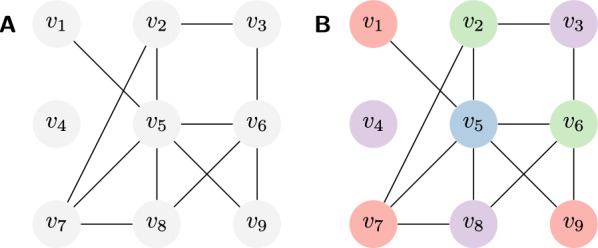
The running example: graph coloring. **A** Example input graph. **B** One valid coloring with 4 colors, corresponding to an assignment of variables to colors (domain values) that satisfies all the inequality constraints along the edges. In our example extension, which minimizes the *feature* counting the different colors in each of its four cycles of length $4$, $(v_2,v_3,v_5,v_6)$, $(v_2,v_5,v_7,v_8)$, $(v_5,v_6,v_7,v_8)$ and $v_5,v_6,v_8,v_9$, this coloring is not optimal (e.g. recolor $v_3$)

### Example 1

(Graph coloring) Let us illustrate this idea by modeling graph coloring as a Constraint Satisfaction Problem (CSP). We use this ‘toy problem’ as our running example to formally introduce our main concepts. For this purpose, we will later extend it beyond constraint satisfaction (introducing some quality of colorings).

Given a graph $G=(V, E)$, see Fig. 1A, the graph coloring problem asks for a vertex labeling by $k$ colors, such that adjacent vertices are colored differently (Fig. 1B). To solve this classical problem in our system, we model it as a CSP, i.e. as a triple of a set of variables, one domain per variable, and constraints. This CSP introduces one variable per vertex, resulting in the set of variables $\{X_1,\dots ,X_{|V|}\}$. Each variable encodes the label of the corresponding vertex, i.e. it takes values from $1$ to $k$, expressed by choosing the domain $D(X_i)=\{1,\dots , k\}$ for each $X_i$. Finally, we define the constraint set consisting of one inequality constraint ${\text {\textsf{NotEquals}}}$ between the variables $X_i$ and $X_j$ for every edge $(i,j)\in E$.

Solving the problem means finding a *valid* assignment of values to variables that satisfies the constraints. Our system supports constraint solving, even if pure constraint solving serves mostly as a basis for further extensions. We can directly express our graph coloring model in Python code.
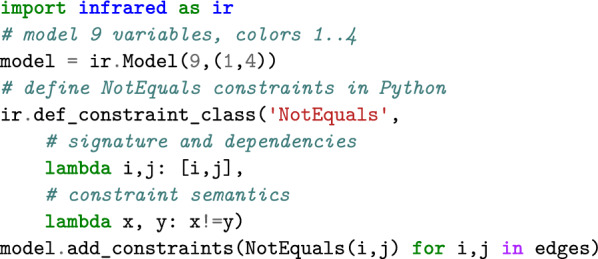


Based on this model, Infrared finds a valid coloring automatically due to its built-in parameterized algorithms in a time that depends on the size of the graph, the number of colors, and the *complexity of G*, i.e. its treewidth. For this purpose, one passes the model to the solver and asks for a valid solution. Since Infrared handles constraint satisfaction and optimization in the same way, its solver is called Optimizer.
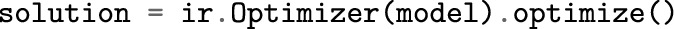


### Extending CSPs by features

Beyond validity, Infrared addresses solution quality in terms of one or several *features*—conceptually, we extend Infrared ’s models from CSPs to feature networks. This allows users of the framework to more naturally model complex problems with multiple objectives, as they commonly occur in bioinformatics. Based on specified features, Infrared is then able to perform tasks such as optimization and weighted sampling.

#### Example 2

As a first feature example, let us pick up graph coloring and additionally minimize the use of different colors in cycles of length $4$ (4-cycles). For this purpose, we specify a feature by imposing one 4-ary function Card for each 4-cycle that counts the different colors in the cycle (set cardinality); the sum of function values defines the value of the feature.

Infrared ’s syntax supports the compositional construction and extension of models. After defining the class of functions Card, we can therefore add them to the previous model.
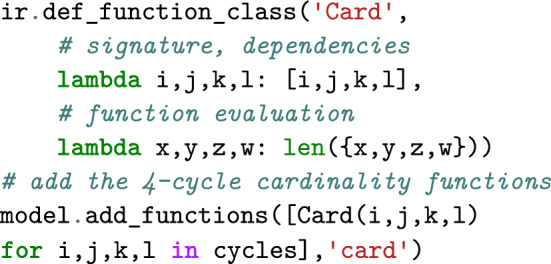


Given this extended model, the solver automatically finds an assignment with optimal evaluation by the feature.
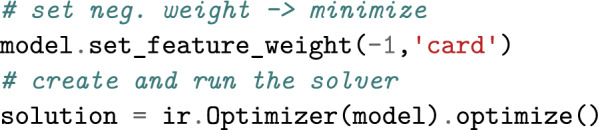


Due to the features, the dependencies between variables become more complex. Where we had a dependency between the variables of each edge in the basic graph coloring model, after the extension, all four variables of each 4-cycle depend on each other due to the functions Card. Infrared ’s solver automatically adapts to this increased complexity of the problem.

### Boltzmann sampling

Once specified by a model, a problem can be *solved* in different ways. In addition to finding optimal solutions, one can just as easily sample assignments from a uniform or Boltzmann distribution controlled by potentially multiple features and their weights.

#### Example 3

Continuing our example, we can generate random uniform colorings from the same model using a different solver.

In statistical mechanics, Boltzmann distributions describe the probabilities of states in a physical system depending on their energy. They are ubiquitous in physics and have numerous applications in bionformatics e.g. for describing the equilibrium of folding molecules [21] or generating energy weighted and near-optimal conformations [22]. Beyond physical interpretation, Boltzmann distributions have applications as general tools, e.g. in heuristic optimization [23], for deriving probabilities in alignments [24, 25] or for targeting properties [15].
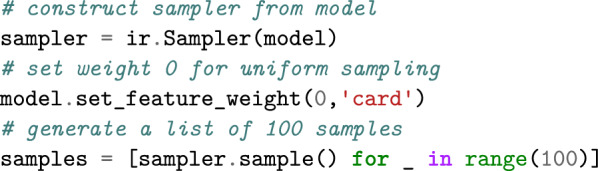


Through the weight, we can control the expected value of the feature in the generated distribution. Setting a nonzero weight causes Infrared to sample from a nonuniform Boltzmann distribution, e.g. setting the weight to $+2$ shifts the expectation to a large cardinality while setting it to $-2$ induces smaller cardinalities.

### Positioning against prior work

As already hinted by the introductory example, Infrared does **not** focus on general constraint solving as performed by constraint programming systems such as Gecode [26]. Adding evaluation to our models ties this work closer to weighted constraint problems or cost networks, with some superficial relations to cost function optimizers such as Toulbar2 [27]. While such systems combine search with forms of constraint consistency, our solving strategies come from the area of constraint processing in constraint networks [12].

As such, our system is tailored to exactly and efficiently solve a specific class of problems, where it can algorithmically profit from a sufficiently tree-like structure (parameterized complexity for the parameter treewidth). This characteristic still allows broad and flexible use of the system, e.g. in bionformatics, where many relevant problems and problem instances have this structure. The capability to solve such bioinformatics problems by complete and exact algorithms with predictable complexity enables specific applications, e.g. it is essential for precisely controlled weighted sampling.

### Overview and contributions

In the next section, we formally define the core concepts of modeling problems in our framework; the models that characterize specific problem instances are formalized as feature networks. Moreover, we precisely state the tasks of optimization and sampling that are solved by the system. In Section “Algorithms for solving feature networks”, we describe the main algorithms to solve these tasks based on the model. Along with the algorithms we explain the underlying prerequisite key concepts of tree decomposition and cluster trees [12]. The given generic, cluster tree elimination-based, algorithms are efficient for fixed treewidth values of the feature network—in other words, they are exponential in the treewidth only. In the second part of the paper, we present several examples of modeling different classic bioinformatics problems as feature networks. Due to the declarative nature of the Infrared system, stating the feature networks is very close to actually implementing these algorithms. To increase the practical value, we put out documented Python code (in the form of Jupyter notebooks) for each application example as supplementary material. Starting with applications to showcase elementary use of Infrared, we move on to advanced topics, including interesting extensions to preceding examples and the targeting of features by multidimensional Boltzmann sampling. Finally, we discuss implications for the use of the system and its application range, as well as future developments within and beyond the framework.

## Feature networks for modeling problems in Infrared

We conceptualize the declarative models of Infrared as *feature networks* (FNs). They are defined as a form of weighted CSP, explicitly distinguishing several real-valued features (instead of only a single or integer-valued score).

### Definition 1

(Feature Network; FN) A feature network (FN) $\mathcal {N}$ is a tuple $(\mathcal {X},\mathcal {D},\mathcal {C},\mathcal {F})$, where

- $\mathcal {X} =\{X_1,\dots ,X_n\}$ is a set of **variables**;
- $\mathcal {D} =\{D_1,\dots ,D_n\}$ is a set of **domains**, one per variable, where each domain is a finite set of integers;
- $\mathcal {C}$ is a finite set of **constraints**;
- $\mathcal {F} =\{F_1,\ldots ,F_\ell \}$ is a set of **features**.

Those networks specify *solutions* to a problem instance as specific assignments of domain values to variables.

### Definition 2

(Assignment) An **assignment**, for a given FN $\mathcal {N}$, is a set of single variable mappings $X_i\mapsto x_i$ such that $X_i$ is a variable of $\mathcal {N}$, $x_i$ is in the domain $D_i$ of $X_i$ and every $X_i$ occurs at most once. An assignment is called **total**, if every variable of $\mathcal {N}$ occurs exactly once; otherwise, it is **partial**.

Given $\mathcal {X} '\subseteq \mathcal {X}$, we denote the set of all **assignments** *x* **of** $\mathcal {X} '$ as $\mathcal {A} _{\mathcal {X} '}$. When $\mathcal {X} '$ (and the order of its variables) is clear, one can write assignments as tuples, e.g. $(x_1,\ldots ,x_n)$ in place of $X_1\mapsto x_1,\dots ,X_n\mapsto x_n$ in the case of a total assignment ($\mathcal {X} '=\mathcal {X}$).

### Example 4

Consider the graph $G=(V,E)$ of Fig. 1. We model graph coloring for *G* and four colors as a feature network $\mathcal {N} _{\text {col}}= (\mathcal {X} _{\text {col}},\mathcal {D} _{\text {col}},\mathcal {C} _{\text {col}},\mathcal {F} _{\text {col}})$. Let us first define $\mathcal {X} _{\text {col}}= \{X_1, \ldots , X_9\}$ and $\mathcal {D} _{\text {col}}= \{D_1,\ldots ,D_9\}$; $D_i=\{1,2,3,4\}$. This specifies one variable $X_i$ for every vertex $v_i$ in the graph and one domain per variable, encoding the colors as integer values.

A total assignment $x=\{X_1\mapsto x_1,\ldots ,\mathcal {X} _9\mapsto x_9\}\in \mathcal {A} _{\mathcal {X} _{\text {col}}}$ describes a coloring where the vertex $v_i\in V$ has color $x_i$.

## Validity of assignments

 To distinguish valid from invalid assignments, we introduce constraints $\mathcal {C}$ that need to be satisfied by valid assignments. In our running example, this allows us to define valid colorings and thus completely specify graph coloring as CSP.

### Definition 3

(Constraint) Given $\mathcal {N} =(\mathcal {X},\mathcal {D},\mathcal {C},\mathcal {F})$, each **constraint** $C\in \mathcal {C}$ is associated with a set of variables $X_{i_1},\ldots ,X_{i_k}\in \mathcal {X}$ and a Boolean function on values $x_{i_1},\ldots ,x_{i_k}$. Given an assignment *x* containing $X_{i_j}\mapsto x_{i_j}$ for all $j\in \{1,\ldots ,k\}$, we **evaluate the constraint** *C* **w.r.t.** *x* by applying the Boolean function to $x_{i_1}, \ldots , x_{i_k}$. The resulting evaluation is denoted *C*(*x*).

$$\begin{aligned} C :\quad&D_{i_1}\times \cdots \times D_{i_k}{} & {} \rightarrow{} & {} \{\textsf{True},\textsf{False} \} \\&(x_{i_1},\dots ,x_{i_k}){} & {} \mapsto{} & {} C(x_{i_1},\dots ,x_{i_k}) \end{aligned}$$

We say *C* is *k***-ary** or has the **arity** *k*. Let ${\text {vars}}(C)=\{X_{i_1},\ldots ,X_{i_k}\}$ denote the **dependency** of *C*. Note that the constraint literature commonly refers to the dependency of a constraint as its **scope**.

We call an assignment $x\in \mathcal {A} _{\mathcal {X} '}$, $\mathcal {X} '\subseteq \mathcal {X}$, **valid** iff all constraints $C\in \mathcal {C}$ with ${\text {vars}}(C)\subseteq \mathcal {X} '$ are **satisfied** (i.e. evaluated to True) by the assignment *x*.

### Example 5

To enforce the neighbor coloring constraint in our example, it is sufficient to add the constraint below for each edge $(v_i, v_j)\in E$

$$\begin{aligned} & c_{{{\text{col}}}} \,{\text{ = }}\,\left\{ {{\textsf{NotEquals}}_{{\left[ {i,j} \right]}} \,\left| {\left( {v_{i} ,\,v_{j} } \right)\, \in \,E} \right.} \right\}{\text{with}}\,\\ & \quad {\textsf{NotEquals}}_{{[i,j]}} \,\left( {x_{i} ,x_{j} } \right)\, = \,\delta \left( {x_{i} \ne x_{j} } \right), \end{aligned}$$

where $\delta (p)$ is the truth value of the expression $p$.

The constraint ${\text {\textsf{NotEquals}}}_{[i,j]}$ determines whether two given colors are distinct. Applying on all edges ensures that a valid assignment is a solution of the graph coloring problem. For example, both assignments $x_{\text {col}}=(1,2,3,3,4,2,1,3,1)$ and $x'_{\text {col}}=(1,2,4,3,4,2,1,3,1)$ satisfy the constraints $\mathcal {C} _{\text {col}}$.

In addition to finding valid assignments, one often wants to distinguish solutions by their quality. In graph coloring, we can e.g. aim for using fewer colors per 4-cycle; this would make $x'_{\text {col}}$ preferable over $x_{\text {col}}$.

## Evaluation of assignments by features

 Each feature $F\in \mathcal {F}$ is a set of *network functions*. In this way, a feature can describe a global property of assignments, in contrast to constraints and network functions which typically act on a small number of variables. This asymmetry is introduced intentionally to allow us to easily control multiple global properties. It specifies an *evaluation* as a sum over the values of the functions in this set; the single functions are defined in the same way as constraints but return real values (instead of Boolean ones).

### Definition 4

(Network Function) Each **network function** $f$ of a feature network is associated with variables $X_{i_1},\ldots ,X_{i_k}\in \mathcal {X}$ and a real-valued function that, given an assignment *x*, maps the values $x_{i_1}, \ldots , x_{i_k}$ to a real number.

$$\begin{aligned} f:\quad&D_{i_1}\times \cdots \times D_{i_k}{} & {} \rightarrow{} & {} \mathbb {R} \\&(x_{i_1},\dots ,x_{i_k}){} & {} \mapsto{} & {} f(x_{i_1},\dots ,x_{i_k}). \end{aligned}$$

Analogous to constraints, the returned value is called the **evaluation of** $f$ **by** *x*, denoted *f*(*x*), and the dependency is ${\text {vars}}(f):=\{X_{i_1},\ldots ,X_{i_k}\}$.

Overloading notation, we define the **(induced) feature evaluation** (of valid assignment *x* by feature *F*) by $F(x) = \sum _{f\in F}f(x)$. To account for multiple features, Infrared combines them linearly.

### Definition 5

(Assignment evaluation) Given a feature network $\mathcal {N} =(\mathcal {X},\mathcal {D},\mathcal {C},\mathcal {F})$ and **feature weights** $\alpha$; $\alpha$ defines respective weights $\alpha _F$ for each feature *F* in $\mathcal {F}$. The evaluation of a valid assignment $x\in \mathcal {A} _\mathcal {X}$ is defined as a linear combination of the feature values w.r.t. $\alpha$.

$$\begin{aligned}E_{\mathcal {N}}(x, \alpha ) = \sum _{F\in \mathcal {F}}\alpha _FF(x).\end{aligned}$$

### Example 6

We can now express our objective in the extended graph coloring problem in terms of a feature. For this purpose, we introduce network functions that each count the different colors in a 4-cycle $(v_i,v_j,v_k,v_l)$ of the example graph,

$$\begin{aligned}{\text {\textsf{Card}}}_{[i,j,k,l]}(x_i,x_j,x_k,x_l) = |\{x_i,x_j,x_k,x_l\}|.\end{aligned}$$

The corresponding feature set is then $\mathcal {F} _{\text {col}} = \{F_{\text {card}}\}$ with

$$\begin{aligned}F_{\text {card}}=\{{\text {\textsf{Card}}}_{[2,3,5,6]},{\text {\textsf{Card}}}_{[2,5,7,8]},{\text {\textsf{Card}}}_{[5,6,7,8]}\}.\end{aligned}$$

In feature network $\mathcal {N} _{\text {col}}=(\mathcal {X} _{\text {col}},\mathcal {D} _{\text {col}},\mathcal {C} _{\text {col}},\mathcal {F} _{\text {col}})$, the two assignment examples $x_{\text {col}}$ and $x'_{\text {col}}$ are evaluated to $E_{\mathcal {N}}(x_{\text {col}},1)=3+4+4=11$ and $E_{\mathcal {N}}(x'_{\text {col}},1)=2+4+4=10$, respectively (for feature weight 1).

Observe that a constraint satisfaction problem (CSP) is a special case of a feature network $(\mathcal {X},\mathcal {D},\mathcal {C},\mathcal {F})$, where $\mathcal {F}$ is empty. Feature networks are one of many possible extensions of CSPs known from the literature [12] that add forms of quality evaluation. For example, cost networks typically contain only a single set of functions, whereas we decided to explicitly distinguish a set of constraints from multiple sets of functions (features).

## Infrared ’s modeling syntax

Recall the code snippets from the introduction. This code implements the feature network $\mathcal {N} _{\text {col}}$ that we formally developed above. As in our formal model description, the definition via Infrared ’s Python interface defines variables and domains, constraint and function types, and sets of constraints and functions. By providing the functionality to add constraints and functions to a model, we support compositional step-by-step construction and even extension of existing models.

Finally, our code examples demonstrate how models are fed to solvers, e.g. ir.Optimizer or ir.Sampler. This allows finding an optimal solution or generating controlled samples from the same model. We formally state the respective Problems 1 and 2 below; these solvers implement the algorithms of Sec. “Algorithms for solving feature networks”.

To keep this article concise, we refer the reader to our online reference and tutorials for syntactic aspects of using Infrared. For further reference, we recommend our coding-oriented introduction in Infrared in a book chapter [28], which focuses on modeling of sequence and RNA design problems. Moreover, recall that we maintain an online archive of documented Infrared application examples (covering all examples of this paper).

## Problem statements

Given a feature network model $\mathcal {N}$, there are two tasks of immediate particular interest: optimization and sampling of the solution space. Our framework addresses both tasks explicitly and solves them automatically based on the specification of $\mathcal {N}$. First, we want to optimize assignments among all valid assignments of $\mathcal {N}$. Concretely, we ask for the assignment that optimizes the evaluation, i.e. the linear combination of the features given specific feature weights $\alpha$.

### Problem 1

(Assignment maximization)

Input: Feature network $\mathcal {N}$, feature weights $\alpha$
Output: Valid assignment $x^*\in \mathcal {A} _\mathcal {X}$ that is maximal w.r.t. $E_{\mathcal {N}}$ and $\alpha$:
  $$x^{*} \, = \,\mathop {{\text{arg}}\,{\text{max}}}\limits_{{\begin{array}{*{20}c} {x \in {\mathcal{A}}_{{\mathcal{X}}} } \\ {x{\text{ is valid}}} \\ \end{array} }} \,E_{{\mathcal{N}}} \left( {x,\alpha } \right).$$

Furthermore, we want to use models to sample valid assignments from a Boltzmann distribution, i.e. each sample should be generated with a probability proportional to the Boltzmann weight of their evaluation w.r.t. a given $\alpha$.

### Problem 2

(Assignment sampling)

Input: Feature network $\mathcal {N}$, feature weights $\alpha$
Output: Valid assignment $x\in \mathcal {A} _\mathcal {X}$ generated with a probability that is proportional to its Boltzmann weight w.r.t. $E_{\mathcal {N}}$ and $\alpha$:
  1 $$\begin{aligned} \mathbb {P}(x) \propto \exp \left( E_{\mathcal {N}}(x, \alpha )\right) . \end{aligned}$$

Unfolding the assignment sampling problem, we realize that it implicitly asks for the **partition function**

$$\begin{aligned}Z_{\mathcal {N}, \alpha }:= \sum _{\begin{array}{c} x\in \mathcal {A} _\mathcal {X} \\ x \text { is valid} \end{array}} \quad \exp \left( E_{\mathcal {N}}(x, \alpha )\right) ,\end{aligned}$$

i.e. the proportionality factor in Eq. 1, such that

$$\begin{aligned}\mathbb {P}(x) = \exp \left( E_{\mathcal {N}}(x, \alpha )\right) / Z_{\mathcal {N},\alpha }.\end{aligned}$$

## Algorithms for solving feature networks

Given a feature network $\mathcal {N}$, Problem 1 asks for an optimal assignment to the variables. Here, the exponentially large assignment space forbids naive approaches. Based on a **tree decomposition** [29] of the network, we employ a form of dynamic programming that decomposes the computation into

- a ‘forward’ optimization phase to determine the optimal evaluation (i.e. only its numerical value), while storing the results of subproblems
- and a subsequent traceback algorithm to obtain an optimal assignment.

Our approach performs the optimization on a tree-like structure, namely, an annotated *tree decomposition* of the network, called the *cluster tree*. Instead of inefficiently searching through all total assignments, it enumerates value combinations of variable subsets at tree nodes and avoids redundant computation by storing the results of subproblems/subtrees; the evaluation of functions and constraints is interleaved with this enumeration. The optimization traverses the tree in bottom-up order; moving top-down in the same tree, based on the (intermediary) results of the first phase, the traceback algorithm identifies one optimal assignment. As such, the approach is a form of **cluster tree elimination (CTE)** [12].

### Sampling resembles optimization

Assignment sampling (Problem 2) can be solved in a remarkably similar way to Problem 1. This task can also be split into two phases, namely, the computation of **partition functions** followed by stochastic traceback. Similar to standard traceback, stochastic traceback constructs solutions by tracing back through the partial results from the forward computation. However, it randomly selects values of variables based on partial partition functions. In this way it finally selects a total assignment from the intended distribution.

To emphasize the parallels between the problems, let us restate optimization as determining

$$\begin{aligned} E_{\max }&= \max _{\begin{array}{c} x\in \mathcal {A} _\mathcal {X} \\ x \text { is valid} \end{array}} \sum _{F\in \mathcal {F}} {\text {\textsf{Id}}}(\alpha _FF(x)) \\ {}&= \max _{\begin{array}{c} x\in \mathcal {A} _\mathcal {X} \\ x \text { is valid} \end{array}} \sum _{F\in \mathcal {F}} \sum _{f\in F}{\text {\textsf{Id}}}(\alpha _Ff(x)) \end{aligned}$$

where ${\text {\textsf{Id}}}$ is the identity function, compared to the partition function

$$\begin{aligned} Z_{\mathcal {N}, \alpha }&= \sum _{\begin{array}{c} x\in \mathcal {A} _\mathcal {X} \\ x \text { is valid} \end{array}} \prod _{F\in \mathcal {F}} \exp \left( \alpha _F F(x)\right) \\ {}&= \sum _{\begin{array}{c} x\in \mathcal {A} _\mathcal {X} \\ x \text { is valid} \end{array}} \prod _{F\in \mathcal {F}} \prod _{f\in F} \exp \left( \alpha _F f(x)\right) . \end{aligned}$$

This breakdown into single network functions suggests that a general scheme can be applied to both problems, which specializes to either problem by the choice of algebra: $(\max , +, {\text {\textsf{Id}}})$ for optimization and $(+, \times , \exp )$ for the partition function (and thus sampling).

### Computation guided by cluster trees

We will define a cluster tree as an annotated tree decomposition of a feature network; it assigns the network functions and constraints to nodes (also called bags or clusters) where they should be evaluated. The computations process these clusters. Here, the tree decomposition determines the processing order. Processing the clusters bottom-up in the forward phase computes a result for the subtree of each cluster. For each cluster, this involves enumerating the assignments of cluster variables, while evaluating the constraints and functions of the cluster as well as previously computed results from the children clusters. The traceback follows the cluster tree top-down, partially re-evaluates the clusters and, on this basis, determines variables.

#### Dependency graphs, tree decompositions, and cluster trees

To properly guide the dynamic programming evaluation, cluster trees must reflect the network dependencies through tree decompositions.

##### Definition 6

(Dependency graph) The **dependency graph of** $\mathcal {N} =(\mathcal {X},\mathcal {D},\mathcal {C},\mathcal {F})$ is the hypergraph $G_{\mathcal {N}}=(\mathcal {X}, \mathcal {E_\mathcal {N}})$, where the hyperedges $\mathcal {E_\mathcal {N}}$ are the dependencies of the constraints and functions:

$$\begin{aligned}\mathcal {E}_\mathcal {N}:= \Big \{{\text {vars}}(C) \mid C\in \mathcal {C}\,\Big \} \cup \Big \{{\text {vars}}(f) \mid f\in \bigcup _{F\in \mathcal {F}} F\,\Big \}.\end{aligned}$$

##### Definition 7

(Tree decomposition, treewidth) A **tree decomposition of a dependency graph** $G_{\mathcal {N}}$ is a pair $(T,\chi )$ of a (rooted) tree $T=(V,E)$ and a node labeling $\chi$ by subsets of variables, i.e. $\chi :V\rightarrow 2^\mathcal {X}$. These subsets are called the **bags** of the tree decomposition.

1. Each variable $X\in \mathcal {X}$ is in at least one bag;
2. For all hyperedge $e\in \mathcal {E}_\mathcal {N}$, there is a node $u\in V$, such that $e\subseteq \chi (u)$;
3. For all variables $X\in \mathcal {X}$, the set $\{u\in V \mid X\in \chi (u)\}$ induces a connected tree.

The **width** of a tree decomposition $(T,\chi )$ is

$$\begin{aligned}\max _{u\in V} |\chi (u)| - 1.\end{aligned}$$

The **treewidth** of a hypergraph is the minimum width among all possible tree decompositions. The **tree decomposition and treewidth of a feature network** $\mathcal {N}$ are the respective tree decomposition and treewidth of its dependency graph $G_\mathcal {N}$.

For a tree decomposition $(T,\chi )$, $T=(V,E)$, consider two nodes $u,v\in V$, where *v* is the parent of *u*. Generally, we assume the tree edges to be oriented toward the root, such that $u\rightarrow v \in E$. We define two sets:

- ${\text {sep}}(u):=\chi (u)\cap \chi (v)$ the **separator set** of shared variables between *u* and its parent; this set describes the variables whose values are passed between parent and child in a tree traversal;
- ${\text {diff}}(u):=\chi (u){\setminus } {\text {sep}}(u)$ the **difference set** between the child and its parent. These are the ‘introduced’ variables by the child; they will be assigned at a child in the top-down traversal of the traceback.

To simplify the traceback step, we require tree decompositions to have empty root and difference sets of size 1 (i.e. $|{\text {diff}}(u)| = 1$ for every child node $u$); we call this property **gentle**. Gentle tree decompositions have exactly one edge per variable. Note that any tree decomposition (as defined above) can be efficiently turned into a gentle decomposition of the same width by inserting additional bags wherever the difference set is too large and contracting edges where no variables are introduced.

**Fig. 2:**
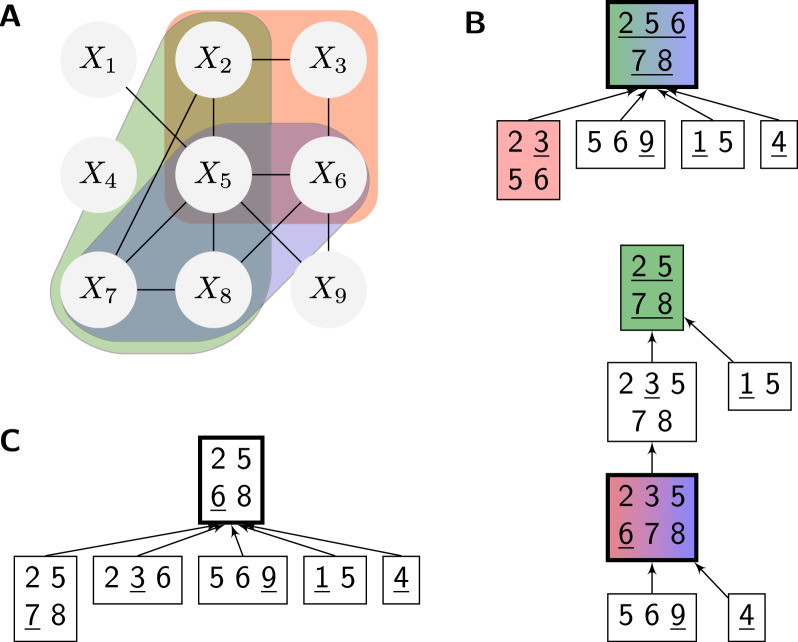
Dependency graph and tree decompositions of the running example (feature network $\mathcal {N} _{\text {col}}$). **A** The dependency graph contains one (binary) edge per dependency due to a constraint ${\text {\textsf{NotEquals}}}\in \mathcal {C} _{\text {col}}$. The dependency hyperedges due to the three network functions ${\text {\textsf{Card}}}\in F_{\text {card}}$ are colored. **B** Two possible tree decompositions of this dependency graph (and therefore $\mathcal {N} _{\text {col}}$). The difference set is underlined in each bag. Solving of the network could be based on either one, but with different run time, which is dominated by the largest bag (bold). Due to their largest bags of size 5 and 6, the two tree decompositions have respective *width* 4 and 5. The bags handling the 4-ary functions are highlighted, where colors correspond to the hyperedges of A. **C** Tree decomposition of the network without 4-ary functions ${\text {\textsf{Card}}}$. The functions don’t allow any tree decomposition with width 3; thus they make the problem more complex

##### Definition 8

(Cluster Tree) A **cluster tree** $(T, \chi , \phi )$ **of an** FN $\mathcal {N} =(\mathcal {X},\mathcal {D},\mathcal {C},\mathcal {F})$ is a tree decomposition $(T=(V,E),\chi )$ of $G_{\mathcal {N}}$ together with an annotation $\phi : V\rightarrow 2^\mathcal {C} \cup 2^\mathcal {F}$; it associates every $f\in \bigcup \mathcal {F}$ and $C\in \mathcal {C}$ to exactly one node $u\in V$ such that ${\text {vars}}(f) \text { and } {\text {vars}}(C)\subseteq \chi (u)$.

We use notations $C\in \phi (u)$ and $f\in \phi (u)$ to refer to the constraints and network functions assigned to node *u* of the cluster tree, respectively. Given a cluster tree, we write $\alpha _f$ to refer to the weight $\alpha _F$ of the feature $F\in \mathcal {F}$ that contains *f*.

In addition to the general cluster tree definition, we assume that constraints and functions are assigned to the lowest possible bag (corresponding to the smallest possible subtree).

#### Evaluation following the cluster tree

For efficiency, Infrared evaluates constraints and network functions as soon as sufficient partial information is available (in contrast to a *generate-and-evaluate* strategy, which would enumerate assignments and evaluate only total assignments).

Recall the notion of *partial assignments* from Definition 2. The evaluation of a constraint (resp. network function) w.r.t. the partial assignment ${\bar{x}}$ is denoted $C(\bar{x})$ (resp. $f(\bar{x})$); it is defined if the assignment assigns all variables of the dependency ${\text {vars}}(C)$ (resp. ${\text {vars}}(f)$). Moreover, the union of partial assignments is well-defined if their variable sets are disjoint; this allows for the composition of larger partial assignments from smaller ones.

##### Example 7

(Partial assignments) Consider the bag $\{X_2,X_5,X_6,X_7,X_8\}$ of Fig. 2B (root of first tree) from the running graph-coloring example with cardinality network functions ${\text {\textsf{Card}}}$. The set $\bar{x}_{\text {col}}=\{X_2\mapsto 2, X_5\mapsto 4, X_6\mapsto 2, X_7\mapsto 1, X_8\mapsto 3\}$ is a partial assignment of the bag’s variables; it lets us evaluate ${\text {\textsf{Card}}}_{[2,5,7,8]}$, ${\text {\textsf{Card}}}_{[2,5,7,8]}(\bar{x}_{\text {col}})=4$, since the bag contains the dependency variables of this function. Consider another partial assignment $\bar{x}'_{\text {col}}=\{X_2\mapsto 2, X_5\mapsto 4, X_6\mapsto 3, X_7\mapsto 1, X_8\mapsto 3\}$; $\bar{x}'_{\text {col}}$ is not valid because ${\text {\textsf{NotEquals}}}_{[6,8]}$ evaluates to False w.r.t. $\bar{x}'_{\text {col}}$.

Given a cluster tree $(T,\chi ,\phi )$ and a node *u* with parent *v*, the forward optimization algorithm successively computes optimal evaluations for subtrees $T_{u}$ below nodes *u* (constituting subproblems of Problem 1).

The **optimal evaluation of subtree** $T_{u}$ is

2 $$\begin{aligned} \max _{\begin{array}{c} \bar{x}\in \mathcal {A} _{\chi (T_{u})}\\ \bar{x} \text { is valid} \end{array}} \sum _{f\in \phi (T_{u})}\alpha _ff(\bar{x}) \end{aligned}$$

where $$\chi (T_{u}):= \bigcup _{c\in T_{u}}\chi (c)$$ and $$\phi (T_{u}):= \bigcup _{c\in T_{u}}\phi (c)$$.

To obtain total subtree evaluations, the algorithm computes and stores *conditional* optimal subtree evaluations, which depend on the partial assignment to ${\text {sep}}(u)$. Thus, they allow decoupling the subtree from the remaining tree.

For a node *u*, these conditional evaluations are computed for all such valid partial assignments, such that they specify network functions $m_{u \rightarrow v}$. We call them **messages** since they are used to pass information from child *u* to parent *v*.

Additionally, define ${\text {diff}}(T_{u}):=\chi (T_{u}){\setminus }{\text {sep}}(u)$ as the set of **variables introduced by** $T_u$.

##### Definition 9

(Conditional optimal subtree evaluation) Let $u$ be a node in the cluster tree $(T,\chi ,\phi )$; denote its parent by *v*. The **conditional optimal subtree evaluation** at *u* under condition $\bar{x}\in \mathcal {A} _{{\text {sep}}(u)}$ is

3 $$\begin{aligned} m_{u\rightarrow v}(\bar{x}) = \max _{\begin{array}{c} \bar{x}'\in \mathcal {A} _{{\text {diff}}(T_{u})}\\ \bar{x}\cup \bar{x}' \text { is valid} \end{array}} \sum _{f\in \phi (T_{u})}\alpha _ff(\bar{x}\cup \bar{x}') \end{aligned}$$

Since the root of *T* is empty, conditional optimal subtree evaluations allow us to directly infer the total evaluation at the root. For every child *u* of the root, ${\text {sep}}(u)$ is empty; moreover, the children of the root do not have any variables in common (due to the definition of tree decomposition). Consequently, we obtain the total evaluation by summing the $0$-ary messages sent to the root from its children

$$\begin{aligned} E_{\max } = \sum _{\text {child } u \text { of root}} m_{u\rightarrow \text { root}}(\varnothing ). \end{aligned}$$

**Fig. 3:**
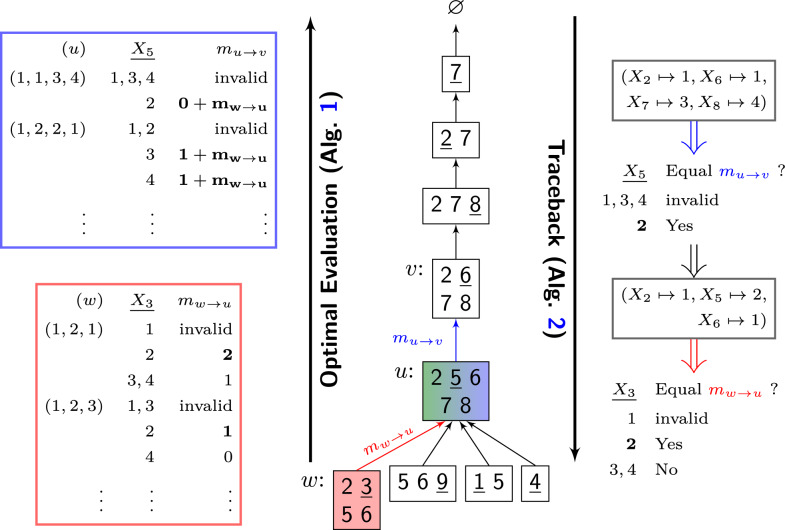
Illustration of the forward optimal evaluation and traceback algorithms (by the running example of graph coloring; Fig. 1). We elaborate steps of the computation guided by the gentle tree decomposition corresponding to Fig. 2B (top). The indices of variables in the difference set are underlined. On the left, we sketch the computation of the messages $m_{w\rightarrow u}$ and $m_{u\rightarrow v}$: For every assignment of the separator set, the algorithm maximizes over assignments of the difference variable (it dismisses invalid assignments); in the computation of $m_{u\rightarrow v}$, it used the already computed message $m_{w\rightarrow u}$. On the right, we show the corresponding computations to assign values to the *underlined* variables during traceback: given an optimal assignment to the variables in *v*, we first infer that $X_5=2$ is an optimal continuation, and finally $X_3=2$

See Fig. 3 for an illustration of the bottom-up computation and the subsequent top-down traceback. Following Proposition 1 each bag $u$ can be processed together with the messages sent to it from its children; thus, we can understand the full computation as bottom-up message passing (Algorithm 1). The notion of message passing stems from more general formulations of CTE on unrooted cluster trees [30]. The algorithm is correct due to the following proposition (shown in Additional file 1).

##### Proposition 1

Let $u\rightarrow v$ be a cluster tree edge and $\bar{x}\in \mathcal {A} _{{\text {sep}}(u)}$ be a partial assignment of ${\text {sep}}(u)$. The conditional optimal subtree evaluations $m_{u\rightarrow v}(\bar{x})$ (Eq. 3) can be recursively computed as

4 $$\begin{aligned} m_{u\rightarrow v}(\bar{x}) = \!\!\!\max _{\begin{array}{c} \bar{x}'\in \mathcal {A} _{{\text {diff}}(u)}\\ \bar{x}\cup \bar{x}' \text { is valid} \end{array}}\! \left[ \!\begin{array}{l} \sum _{f\in \phi (u)} \alpha _ff(\bar{x}\cup \bar{x}') \\ + \sum _{c\text { child of }u} m_{c\rightarrow u}(\bar{x}\cup \bar{x}') \end{array}\!\right] . \end{aligned}$$

*Algorithms for partition functions and sampling* As argued, the computation of partition functions (Problem 2) follows the same algorithmic structure, changing the algebra in Algorithm 1 from $(\max , +, {\text {\textsf{Id}}})$ to $(+, \times , \exp )$ and setting the initial value of $t$ to $0$. Consequently, the partition function $Z_{\mathcal {N},\alpha }$ is obtained by multiplying all $0$-ary messages sent to the root.

Analogous to partial optimal evaluations, the modified Algorithm 1 with $(+, \times , \exp )$-algebra computes partial partition functions.

##### Definition 10

(Conditional subtree partition functions) Let *u* be a node in a cluster tree $(T,\xi ,\phi )$, where *v* is its parent. The **conditional partition function** at *u* under condition $\bar{x}\in \mathcal {A} _{{\text {sep}}(u)}$ is

5 $$\begin{aligned} m_{u\rightarrow v}(\bar{x}) = \!\!\sum _{\begin{array}{c} \bar{x}'\in \mathcal {A} _{{\text {diff}}(T_{u})}\\ \bar{x}\cup \bar{x}' \text { is valid} \end{array}} \prod _{f\in \phi (T_{u})}\!\exp (\alpha _f f(\bar{x}\cup \bar{x}')) \end{aligned}$$

for all $$\bar{x}\in \mathcal {A} _{{\text {sep}}(u)}.$$

Partition functions are computed by a recursive algorithm analogous to 1; its correctness is stated in analogy to Proposition 1 (shown in Additional file 1).

##### Proposition 2

Let $u\rightarrow v$ be a cluster tree edge and $\bar{x}\in \mathcal {A} _{{\text {sep}}(u)}$ be a partial assignment of ${\text {sep}}(u)$. The conditional subtree partition functions (Eq. 5) can be recursively computed as

6 $$\begin{aligned} m_{u\rightarrow v}(\bar{x}) = \!\sum _{\begin{array}{c} \bar{x}'\in \mathcal {A} _{{\text {diff}}(u)}\\ \bar{x}\cup \bar{x}' \text { is valid} \end{array}}\! \left[ \!\begin{array}{l} \prod _{f\in \phi (u)} \exp (\alpha _ff(\bar{x}\cup \bar{x}')) \\ \times \prod _{c\text { child of }u} m_{c\rightarrow u}(\bar{x}\cup \bar{x}') \end{array}\!\right] \end{aligned}$$

with $\alpha _f$ as in Prop. 1.

### Traceback

Once all messages of partial optimal score are computed by Algorithm 1, the optimal assignment is obtained by a traceback traversing the cluster tree top-down in preorder (Algorithm 2). At each edge $u\rightarrow v$, an optimal assignment of the variables in the parent *v* is known. Infrared then determines the optimal assignment to the difference variables (in the singleton set ${\text {diff}}(u)$) such that the evaluation of bag *u* equals the message sent to the parent bag $v$. Let $x$ be the partial optimal assignment determined thus far in the algorithm (assigning the variables of *v*); the algorithm searches through $\bar{x}\in \mathcal {A} _{{\text {diff}}(u)}$ and selects one assignment $\bar{x}$ that yields the optimal message. This choice is optimal, such that the algorithm can continue its top-down traversal after updating *x* by $x\cup \bar{x}$.

For sampling, Infrared performs a stochastic traceback (Algorithm 3), requiring the messages from the computation of the partition function. Whereas the general structure resembles that of optimal traceback, at each edge $u\rightarrow v$ the algorithm randomly chooses a tracking value $t$ uniformly from the range $0$ and $m_{u\rightarrow v}$. While iterating through the possible assignments for ${\text {diff}}(u)$, $t$ is gradually decreased with partial Boltzmann factors. The value of the difference variable is selected once $t$ becomes negative. This selects the value following the desired Boltzmann distribution. We show the following correctness claim in Additional file 1.

#### Proposition 3

Algorithm 3 solves Problem 2 by sampling assignments from the Boltzmann distribution of Eq. (1).

**Algorithm 1:**
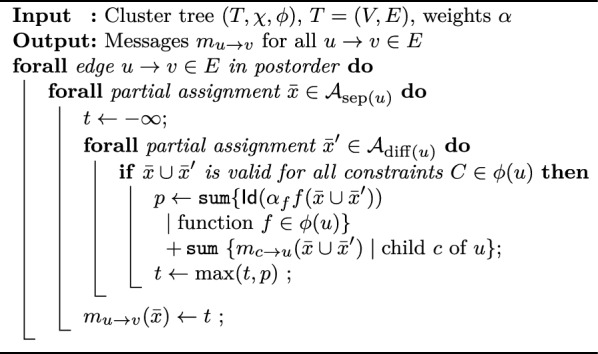
Optimal evaluation

**Algorithm 2:**
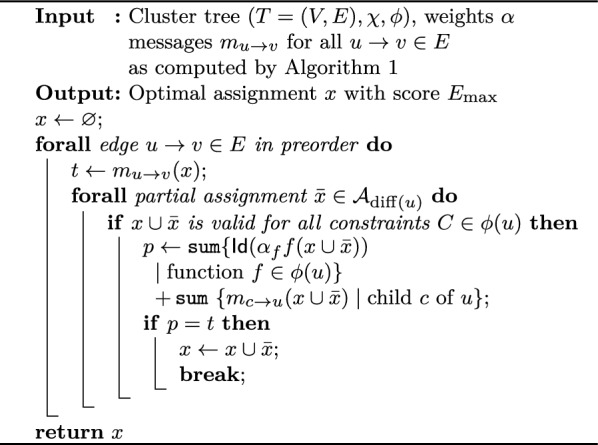
Optimal traceback

**Algorithm 3:**
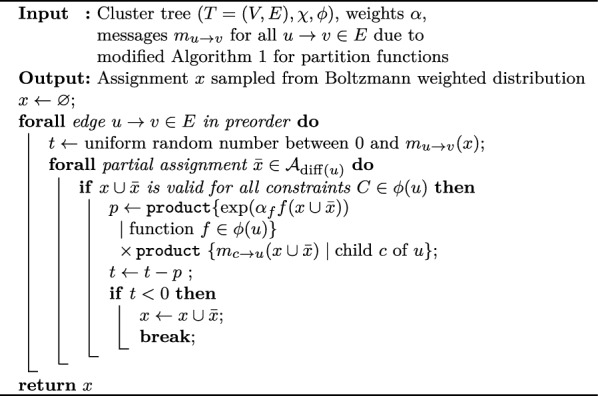
Stochastic traceback: sampling

### Computational complexity

Note that while computational complexities can be interpreted as corollaries from CTE [12], we rephrase the arguments adapted to our concrete algorithms.

For a feature network $\mathcal {N} =(\mathcal {X},\mathcal {D},\mathcal {C},\mathcal {F})$, we state complexities in terms of additional parameters: the **largest domain size** $d:=\max _{D\in \mathcal {D}}|D|$, the **number of variables** $\mathfrak {n}:=|\mathcal {X} |$, and the total **number of constraints and network functions** $\mathfrak {m}:=|\mathcal {C} |+\sum _{F\in \mathcal {F}}|F|$. Let $w$ denote the **treewidth** of dependency graph $G_{\mathcal {N}}$. Furthermore, we assume that single constraints and network functions are evaluated in constant time. We will see later from the applications in Sections “Applications to concrete bionformatics problems” and “Model extensions and advanced topics” the assumption holds in practice.

#### Proposition 4

Algorithm 1 takes $\mathcal {O}\left( d ^{w +1} \cdot (\mathfrak {m} + \mathfrak {n}) \right)$ time and $\mathcal {O}\left( d ^w \cdot \mathfrak {n} \right)$ space.

#### Proof

Algorithm 1 computes one message $m_{u\rightarrow v}$ for each edge $u\rightarrow v$ of the tree decomposition of $\mathcal {N}$. In every child bag *u*, the algorithm computes message values for each assignment of the variables in ${\text {sep}}(u)$, for each value optimizing over assignments of ${\text {diff}}(u)$. In every iteration, it evaluates the constraints and functions in $\phi (u)$, as well as the messages from the children.

We thus bound the computation by

$$\begin{aligned}&\sum _{u\rightarrow v}\ \prod _{x_i\in {\text {sep}}(u)}\!|\mathcal {D} _{i}|\,\cdot \!\prod _{x_i\in {\text {diff}}(u)}\!|\mathcal {D} _i|\,\cdot (|\phi (u)|+n_u), \end{aligned}$$

where $n_u$ counts the children of u. Since ${\text {sep}}(u)$ and ${\text {diff}}(u)$ are disjoint and contain exactly the variables of in the bag *u*, there are at most $d^{w +1}$ iterations per bag. We relax the bound to

$$\begin{aligned}&d ^{w +1}\cdot \sum _{u\rightarrow v} (|\phi (u)|+n_u) \end{aligned}$$

Every constraint and function is evaluated in the iterations of exactly one bag *u*; thus, we can amortize the contributions due to $\sum _{u\rightarrow v} |\phi (u)| = \mathfrak {m}$. Moreover, every message from a child is accessed (in constant time) in the iterations of exactly one bag; we can thus amortize due to $\sum _{u\rightarrow v} n_u = |X|$. This lets us simplify the last bound further to derive the claim on the time complexity.

Concerning space, the algorithm stores a message at each edge of the tree decomposition. Per edge $u\rightarrow v$, this takes space $\mathcal {O}(d ^{{\text {sep}}(u)})$. This bounds the space by $\mathcal {O}(d ^s \cdot |E|)$, where $s=\max _u {\text {sep}}(u)$. For gentle tree decompositions, $|E|=|X|$ and $s\le w$, showing the claim. $\square$

#### Proposition 5

Algo. 2 runs in $\mathcal {O}\left( d \cdot (\mathfrak {m} + \mathfrak {n}) \right)$ time.

#### Proof

For each edge $u\rightarrow v$ of the tree decomposition of $\mathcal {N}$, the task is to determine the best assignment for variables in ${\text {diff}}(u)$, given that variables in ${\text {sep}}(u)$ are already assigned (as guaranteed by the iteration in preorder). Deciding if an assignment is valid requires computing constraints, while scoring them requires computing network functions (each in constant time due to our assumption).

It is also required to sum up $n_u$ messages $m_{c\rightarrow u}$ for *c* children of *u*, where $n_u$ denotes the number of children. Given that $|{\text {diff}}(u)|=1$ in a gentle tree decomposition, we obtain as an upper bound of time complexity in Algorithm 2:

$$\begin{aligned}&\sum _{u\rightarrow v} \ \prod _{x_i\in {\text {diff}}(u)}\!|\mathcal {D} _i| \,\cdot (|\phi (u)|+n_u) \\&\le d \sum _{u\rightarrow v} (|\phi (u)| + n_u) \le d (\mathfrak {m} +\mathfrak {n}). \end{aligned}$$

$$\square$$

Note that the complexity results for optimization and optimal traceback directly apply to partition function computation and stochastic traceback, which evaluate exactly the same numbers of constraints, functions and messages.

### Complexity analysis for nonuniform domain sizes

For nonuniform domain sizes, the previous analysis can strongly overestimate the complexity (assuming the worst-case maximum domain size *d* for all variables). In several of our application examples, we can tighten the analysis considering that $\mathcal {X}$ is composed of two (analogously extensible to several) ‘series’ of variables in the way

$$\begin{aligned}\mathcal {X}=\{X_1,\dots ,X_{n_X},Y_1,\dots ,Y_{n_Y}\}\end{aligned}$$

with respective maximum domain sizes $d _X$ and $d _Y$. For a given tree decomposition, we can define subset widths $w _X$ and $w _Y$ as the maximum number of respective *X* and *Y* variables in a bag minus 1.

Then, we bound more tightly as follows:

$$\begin{aligned}&\sum _{u\rightarrow v}\ \prod _{x_i\in {\text {sep}}(u)}\!|\mathcal {D} _{i}|\,\cdot \!\prod _{x_i\in {\text {diff}}(u)}\!|\mathcal {D} _i|\,\cdot (|\phi (u)|+n_u)\\&\le d _X^{w _X+1}\cdot d _Y^{w _Y+1}\cdot \sum _{u\rightarrow v} (|\phi (u)|+n_u)\\&\le d _X^{w _X+1}\cdot d _Y^{w _Y+1}\cdot (\mathfrak {m} + \mathfrak {n}). \end{aligned}$$

#### Corollary 1

The runtime of Algorithm 1 is in

$$\begin{aligned}\mathcal {O}\left( d _1^{w _1+1}\cdots d _k^{w _k+1} \cdot (\mathfrak {m} + \mathfrak {n}) \right) ,\end{aligned}$$

given a feature network where $\mathcal {X}$ is a disjoint union of subsets $\mathcal {X} _1,\dots ,\mathcal {X} _k$ and a tree decomposition $(T,\chi )$, where $w _i = \max _{v\in T} |\{X \in \mathcal {X} _i \mid X \in \chi (v)\}|$ are the respective subset widths of $\mathcal {X} _1,\dots ,\mathcal {X} _k$ w.r.t. $(T,\chi )$.

It becomes apparent that tree decompositions with minimal width do not necessarily yield best performance in this context (e.g. [31]). We can take a shortcut in special cases, where variables $X_i$ and $Y_i$ for the same $1\le i \le n$ ($n=n_X=n_Y$) depend on each other. Then, one can collapse the nodes of $X_i$ and $Y_i$ in the dependency graph, generate a standard tree decomposition optimizing its width $w '$, and infer a tree decomposition of the original dependency graph with $w _X=w _Y=w '$.

### Parameterized complexity classes

Based on our complexity results (Sec. “Computational complexity”), the Optimization (Prob. 1) and Sampling (Prob. 2) can be solved efficiently in the *input size* *n* at fixed values of the treewidth. Assuming that the number of variables and number of edges is on the order of *n*, i.e. $\mathfrak {n}+\mathfrak {m}\in \mathcal {O}(n)$, the input-dependence of the maximum domain size *d*, $d\in \mathcal {O}(1)$ or $d\in \mathcal {O}(n)$, determines the theoretical parameterized complexity class.

For problems parameterized by *k*, one distinguishes the class **FPT** (**fixed parameter tractable**), where problems can be solved in time $f(k)n^{\mathcal {O}(1)}$ for some computable function *f*, from the class **XP** with a bound of $f(k)n^{g(k)}$ [9, 14] for some computable functions *f*, *g*. The latter class strictly includes the former. **XP** algorithms are also called **slicewise polynomial**, having polynomial complexity for each fixed value of the parameter.

For constant *d*, it follows that solving in Infrared is in the class **FPT** parameterized by the treewidth of the dependency graph. This is the case for the presented applications to RNA design, where the domain size is the number of nucleotides, i.e. typically 4. In our applications to pseudoknotted RNA alignment, the domain size *d* is in $\mathcal {O}(n)$; consequently, we obtain an **XP** solving algorithm.

### Computing tree decompositions

The problem of computing a tree decomposition of minimal treewidth for an input graph/network is NP-hard [32]. However, multiple heuristics [13] and even efficient exact solvers [33] have been designed, motivated by the wide applicability of treewidth-based methods.

From a theoretical perspective, treewidth is FPT to compute, albeit with a prohibitive complexity of $2^{O(w ^3)}$ [34]. A $4\cdot w +4$ approximation in $O(8^{w}\cdot w ^2 \cdot |\mathcal {X} |^2)$ is also possible [14]. Both of these results guarantee that FPT results remain FPT when including the computation of a tree decomposition prior to applying Algorithm 1 and 2. However, the actual complexity may be affected, becoming the worst of the two.

Despite these theoretical results, virtually all treewidth-based implementations, including Infrared, use the beforementioned heuristics or solvers to compute tree decompositions.

## Applications to concrete bionformatics problems

### Network parsimony

**Fig. 4:**
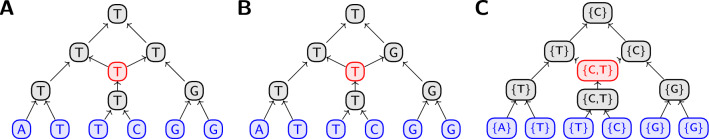
Example phylogenetic network. Optimal solutions for **A** hardwired parsimony. **B** softwired parsimony. **C** parental parsimony; nodes are labeled by character sets. The input for these problems consists of the network and the labels of only the leaves (blue).The other labels are inferred. The example contains one reticulation node (red)

#### ***Parsimony for phylogenetic reconstruction***

 For inferring phylogenies, one of the central missions of bioinformatics, parsimony methods determine the most parsimonious explanations for evolutionary relationships. In the classical small parsimony problem the relation between *n* taxa is given as their phylogenetic tree. The leaves are labeled by ‘characters’, i.e. the taxa, and we ask for a labeling of the internal nodes such that the number of label differences over all tree edges is minimized. However, due to *reticulate evolution*, where lineages can be influenced by two or more ancestors, many real phylogenies are better represented by phylogenetic networks than trees [35]. This model captures diverse phenomena such as hybrid speciation, horizontal gene transfer, and allopolyploidity due to sexual recombination. While tree parsimony has well-established polynomial-time solutions [36, 37], network parsimony is a topic of current algorithmic research. For example, Scornavacca and Weller [5] present artfully hand-crafted fixed-parameter tractable (FPT) algorithms for three variants of network parsimony. We will discuss modeling network parsimony directly in Infrared and, in this way, immediately obtain FPT solutions.

##### Definition 11

[Phylogenetic Network] A **phylogenetic network** is a rooted, connected directed acyclic graph $G = (V,E)$. Edges point from **children** to their **parents**. The unique **root** $r\in V$ is the only node without parents; the **leaves** $L \subseteq V$ are the nodes without children. **Reticulation nodes** have more than one parent.

**Hardwired parsimony** can be seen as a direct extension from tree to network parsimony that minimizes a parsimony score summing over all network edges, **softwired parsimony** inherits—in the case of multiple parents—only from the most favorable one, and **parental parsimony** allows embedding of different lineages in the network (one parent per allele) to cover allopolyploidy [5] (Fig. 4).

In this text, we describe in detail the modelings of hardwired and softwired parsimony. For Infrared models of all three variants of network parsimony, we refer to our online documentation.

##### Problem 3

[Hardwired network parsimony]

Input: Phylogenetic network $G=(V,E)$ with leaves *L*, set of characters $\Sigma$, and leaf labeling $\phi : L\rightarrow \Sigma$.
Output: Minimal parsimony score ${\text {PS}}^{*}_\text{hw}$ and corresponding labeling $\psi :V\rightarrow \Sigma$, where
  $$\begin{aligned} {\text {PS}}^{*}_\text{hw}= \min _{\begin{array}{c} \hbox { labeling}\ \psi \\ \forall v\in L: \psi (v)=\phi (v) \end{array}} \sum _{(u,v)\in E} {\text{d}}(\psi (u),\psi (v)), \end{aligned}$$
  here limiting ourselves, for simplicity, to distance $${\text {d}}(x,y)={\left\{ \begin{array}{ll}1&{}x\ne y\\ 0&{}x=y.\end{array}\right. }$$

#### ***Infrared network model***

We model labellings as assignments, i.e. we use one variable $X_i$ per node of *G*, whose value encodes its label, i.e. the domain of internal nodes is $\Sigma$, while the domain of leaf variables is restricted by the input labeling $\phi$. We can thus specify the variables and domains of the feature network $\mathcal {N} _{\text {hw}}=(\mathcal {X} _{\text {hw}},\mathcal {D} _{\text {hw}},\mathcal {C} _{\text {hw}},\mathcal {F} _{\text {hw}})$, which models Problem 3:

- $$\mathcal {X} _{\text {hw}}=\{X_1,\ldots ,X_{|V|}\}$$
- $${\mathcal{D}}_{{{\text{hw}}}} \, = \,\left\{ {D_{1} , \ldots ,D_{{|V|}} } \right.\,,$$ where $$D_i={\left\{ \begin{array}{ll} \{\phi (i)\} &{} v_i\in L\\ \Sigma &{} \text {otherwise} \end{array}\right. }$$

On this basis, we impose constraints and functions. In this case, there are no constraints (all constraints are expressed by restricting the domains, such that all assignments are valid labellings). To express the score (by a set of network functions), we introduce the network function ${\text {\textsf{Distance}}}_{[i,j]}$ for the variables $X_i$ and $X_j$ is defined to encode the distance $d(x_i,x_j)$ between their values in an assignment *x*. We finalize the model by

- $$\mathcal {C} _{\text {hw}}=\{\}$$
- $$\mathcal {F} _{\text {hw}}=\{F_{\text {hwd}}$$} with feature
  $$\begin{aligned}F_{\text {hwd}}=\{{\text {\textsf{Distance}}}_{[X_i,X_j]} \mid (v_i,v_j) \in E\}.\end{aligned}$$

To implement and solve the problem in Infrared, it suffices to *translate* the model to Infrared syntax and call its optimizer. According to Proposition 4, the framework determines a most parsimonious solution in time complexity $\mathcal {O}((|E|+ |V|)\cdot |\Sigma |^{w +1})$ in the treewidth $w$ of the input network $G=(V,E)$. For this corollary observe that the dependency graph of the modeled feature network is exactly the input network; moreover the model has |*E*| functions and |*V*| variables with maximum domain size $d=|\Sigma |$; functions are computed in constant time.

#### ***Beyond hardwired network parsimony***

The problem of **softwired network parsimony** redefines the score of hardwired parsimony, such that it asks for

$$\begin{aligned} {\text {PS}}^{*}_\text{sw}= \min _{\begin{array}{c} \hbox { labeling}\ \psi \\ \forall v\in L: \psi (v)=\phi (v) \end{array}} \sum_{u\in V}\min _{v\in {\text{parents}}(u)} {\text{d}}(\psi (u),\psi (v)), \end{aligned}$$

where ${\text {parents}}(u)$ denotes the set of parents of *u*. This does not change the behavior at nonreticulation nodes, but offers a choice in the case of reticulation nodes.

Here, we restrict our model to **binary networks**, where nodes can have up to two children and up to two parents. Then, starting from the hardwired model, we enable this choice by adding a Boolean selector variable $Y_i$ for every reticulation node $v_i$. The distance to the left parent is counted if $Y_i=0$; to the right parent, if $Y_i=1$. Then, we replace the distance network functions by special variants at all edges between a reticulate child *u* and one of its parents *v*; feature $F_{hwd}$ is substituted by

$$\begin{aligned} F_{swd} =\,\{{\text {\textsf{RDistance}}}_{[X_i,X_j,Y_i;r]} \mid i, j \in (v_i,v_j)\in E,\\ {}\quad v_i \,\text {is a reticulation node}, \\ {}\quad r=1\, \text {if}\, v_j \,\text {is right parent of}\, v_i \,\text {else 0}\} \\\cup \{{\text {\textsf{Distance}}}_{[X_i,X_j]} \mid i, j \in (v_i,v_j)\in E,\\ {}\qquad v_i \,\text {is not a reticulation node}\}, \end{aligned}$$

where *r* controls the selection, i.e.

$$\begin{aligned}&{\text {\textsf{RDistance}}}_{[X_i,X_j,Y_i;r]}(x_i,x_j,y_i) \\&\qquad = {\left\{ \begin{array}{ll} {\text {\textsf{Distance}}}_{[X_i,X_j]}(x_i,x_j) &{}\hbox { if}\ y_i=r\\ +\infty &{}\text {otherwise.} \end{array}\right. } \end{aligned}$$

To improve over the bound of Proposition 4, we follow Section “Complexity analysis for nonuniform domain sizes”. For the purpose of a conservative worst case complexity analysis, consider a tree decomposition of the dependency graph (which is equivalent to the input network). Now, we modify the problem by adding variables $Y_i$ as well to all the nonreticulation nodes. A tree decomposition of the modified problem can now be obtained by complementing all $X_i$ by corresponding $Y_i$. The subset widths $w _X$ and $w _Y$ are then equal to the original treewidth $w$. Therefore, by Cor. 1, we obtain the complexity $\mathcal {O}( |\Sigma |^{w +1}2^{w} (|V|+|E|) )$ for solving softwired network parsimony.

#### ***Discussion***

 Scornavacca and Weller [5] present algorithms for hardwired, softwired, and parental network parsimony with respective complexities of $\mathcal {O}(|\Sigma |^{w +1}|E|)$, $\mathcal {O}(|\Sigma |^{w}(3^w |\Sigma | |V| + |E|)$, and $\mathcal {O}( 6^{w |\Sigma |} 4^{w \log (c)} |E| )$ (after obtaining the tree decomposition). In the hardwired case, we obtain the same complexity out-of-the-box.

In the case of *softwired complexity* for the special case of binary networks, we even obtain a complexity with a better treewidth dependence. To show this, given $|E|<2|V|$ under the assumption of binary networks, one simplifies our result to $\mathcal {O}(|\Sigma |^{w +1}2^{w}|V|)$ and theirs to $\mathcal {O}(|\Sigma |^{w +1}3^{w}|V| + |\Sigma |^w 2|V|)=\mathcal {O}(|\Sigma |^{w +1}3^w |V|)$.

We refer to our accompanying notebook for the case of *parental parsimony*. There, we provide a model that induces an efficient solution whenever the treewidth of the feature network remains bounded. In this case, the feature network simply consists of the input network, augmented by ternary constraints at reticulation nodes. Consequently, while obtaining an FPT algorithm even for this complex parsimony problem, we cannot directly compare its complexity to [5].

### RNA design

**Fig. 5:**
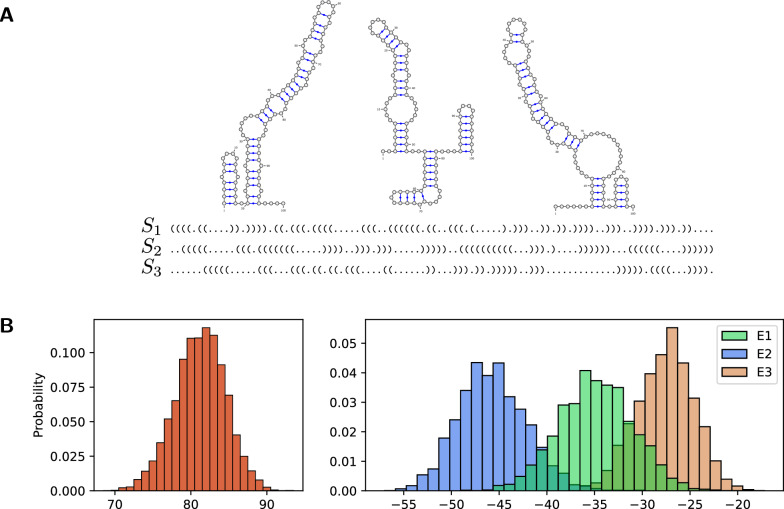
RNA multitarget design. **A** Three target RNA secondary structures of length 100 as 2D plots (by VARNA [38]) and dot-bracket strings; taken from a multitarget design benchmark set [39]. **B** Histograms of the features G **C** content (left), and the Turner energies (kcal/mol) of the three targets (right) in 5000 sequences sampled from the multitarget design model $\mathcal {N} _{\text {design}}$ at weight -5 for every feature. One can observe that (1) equal weights lead to different mean energies for the targets; (2) strong control of the G C weight is required to avoid extreme G C content for stable designs. To automate the calibration of weights (and target specific feature value combinations), we suggest multidimensional Boltzmann sampling in Section “Multidimensional Boltzmann sampling”

Designing biomolecules for specific biotechnological or medical applications is typically an interdisciplinary endeavor combining experimentation and computational design. On the computational side this calls for flexible, extensible systems that can express and efficiently cope with various constraints and objectives—a paradigmatic playing field for our framework (see our treatment in [28]). A challenging, computationally hard subproblem in this area is the design of RNA sequences that fold into multiple target structures. The Infrared framework generalizes the FPT algorithm of our earlier work RNARedprint [16]—a method to generate RNA sequences *w*, words over $\texttt {A}, \texttt {C}, \texttt {G}, \texttt {U}$ targeting the energies of multiple structures and specific G C content. The latter is defined as the amount of G and C characters, denoted $\#\texttt {G} \texttt {C} (w)$. Here, we model the core problem of RNARedprint directly as a feature network, which allows its implementation in Infrared.

#### Definition 12

[RNA secondary structure] A **secondary structure** $S$ of length $n$ is a set of **base pairs**, i.e. pairs (*i*, *j*) of sequence positions, $1\le i< j\le n$. Secondary structures are required to be **free of base triplets**, i.e. every base $1\le i\le n$ is involved in at most one base pair. A secondary structure *S* is called **crossing** iff there are pairs $(i,j), (k,l)\in S$, such that $i<k<j<l$; otherwise, it is **noncrossing**.

*Multitarget design sampling* Given one or multiple noncrossing RNA secondary structures as *targets* (Fig. 5A), we consider the problem of controlled sampling of designs (i.e. RNA sequences) from a Boltzmann distribution governed by the thermodynamic energies of the targets and the G C content, whose respective influence is controlled by weights (Fig. 5B).

#### Problem 4

[Multitarget RNA sequence sampling] Given are *k* target structures, i.e. noncrossing secondary structures $S_1,\dots ,S_k$ of length $n$, together with weights $\alpha _1,\dots ,\alpha _k$ and $\alpha _{\texttt {G} \texttt {C}}$. We ask for *r* RNA sequences of length *n* such that for each sequence $s$

$$\begin{aligned}\mathbb {P}(s) \propto \exp \left( \alpha _{\texttt {G} \texttt {C}}\cdot \#\texttt {G} \texttt {C} (s)\right) \cdot \prod _{\ell =1}^k \exp \left( \alpha _\ell \cdot E(s, S_\ell )\right) \end{aligned}$$

with $E(s, S_\ell )$ is the free-energy of the sequence $s$ folding into the structure $S_\ell$.

*Constraints and functions* In common energy models of RNAs, such as the nearest neighbor model [40], all base pairs must be **canonical**, i.e. in

$$\begin{aligned}\mathcal {B}=\{(\texttt {A},\texttt {U}), (\texttt {C}, \texttt {G}), (\texttt {G},\texttt {C}), (\texttt {G}, \texttt {U}), (\texttt {U}, \texttt {A}), (\texttt {U},\texttt {G})\}.\end{aligned}$$

Otherwise, the energy $E(s, S)$ is infinite. This imposes hard constraints on the solutions of our design problem; in [16], we proved that these constraints make even the counting of valid solutions (with implications on controlled sampling) #P-hard.

In our model, in line with [16], we express a relatively simple energy function $E(s,S)$, namely

$$\begin{aligned}E_{bp}(s,S) = \sum _{(i,j)\in S}{\text {\textsf{BPEnergy}}}(s _i, s _j)\end{aligned}$$

where ${\text {\textsf{BPEnergy}}}: \mathcal {B}\rightarrow \mathbb {R}$ is a function assigning values to single base pairs. Note that we empirically demonstrated the direct use of this simple energy model for design sampling [16] (apart from being extensible to more accurate models). This is in remarkable contrast to structure prediction, which for relevant accuracy relies on models that assign energies to stabilizing and destabilizing loops [40]. Figure 5B shows that sampling based on the simple base pair model can produce controllable concentrated distributions with regard to Turner energies [41]. This effect is studied in more depth in [16].

*Feature network for design* We express Problem 4 as a feature network and use Infrared to solve it. The FN $\mathcal {N} _{\text {design}}$ is composed of

- $$\mathcal {X} _{\text {design}}=\{X_1,\ldots ,X_n\}$$;
- $$\mathcal {D} _{\text {design}}=\{\texttt {A},\texttt {C},\texttt {G},\texttt {U} \}^n$$;
- $$\mathcal {C} _{\text {design}}=\{{\text {\textsf{BPCompl}}}_{[i,j]} \mid (i,j)\in \bigcup _\ell S_\ell \}$$;
- $\mathcal {F} _{\text {design}}=\{F_{gc},F_{1},\dots ,F_{k}\}$ with features $F_{{gc}} \, = \,\left\{ {GC_{{\left[ i \right]}} \,\left| {i\, \in \,\left[ {1,\,n} \right]} \right.} \right\}$ and $F_{\ell }=\{{\text {\textsf{BPEnergy}}}_{[i,j]} \mid (i,j)\in S_\ell \}$ ($1\le \ell \le k$).

The constraint ${\text {\textsf{BPCompl}}}_{[i,j]}(x_i,x_j)$ is $\textsf{True} \text { if } (x_i,x_j)\in \mathcal {B}$; it ensures that $(i,j)$ is a canonical base pair in the design *w*. The network functions ${\text {\textsf{BPEnergy}}}_{[i,j]}$ and ${\text {\textsf{GC}}}_{[i]}$ decompose the global properties, energy and G C content, into their local contributions from base pairs or bases. To evaluate the assignment, feature $F_{gc}$ has a weight of $\alpha _{\texttt {G} \texttt {C}}$ and each feature $F_\ell$ has $\alpha _\ell$ for $\ell \in [1,k]$.

*Efficient solving in Infrared* To randomly generate *r* designs, sampled exactly from the defined distribution of Problem 2, we encode $\mathcal {N} _{\text {design}}$ as an object of the class infrared.Model and pass it to Infrared ’s sampler infrared.Sampler, which is then asked *r*-times to return a sample. The efficiency of sampling depends exponentially on the complexity of the graph $G_{\text {design}}=(\{1,\dots ,n\},\bigcup _{\ell =1}^k S_\ell )$, which combines all the dependencies between sequence positions due to the target structures.

#### Corollary 2

Infrared ’s engine solves Problem 4 in $\mathcal {O}((n+m)\cdot 4^{w}) + r\cdot 4(n+m))$ time and $\mathcal {O}(n\cdot 4^{w})$ space, where $m=n+2\sum _{\ell =1}^k|S_\ell |$, i.e. the total number of functions and constraints, and $w$ is the treewidth of $G_{\text {design}}$.

*Discussion* Multitarget design well showcases the advantages due to a declarative system. Thus, we quickly developed RNARedprint v2 with extended functionality and improved performance compared to our original C++ implementation of RNARedprint [16]. Notably, based on the presented model, this reimplementation has identical computational complexity (Cor. 2).

As expected, the declarative modeling framework in Python strongly facilitated the reimplementation and extension. The performance improvements (Fig. 11A) can be attributed to Infrared ’s systematic Python/C++ hybrid design, which allowed us to better optimize its generic computational engine.

Our Jupyter notebook for multitarget design shows that RNARedprint ’s targeted sampling functionality can be coded in less than 100 lines of Python. Due to Infrared, this code is extensible and adaptable and makes the functionality well accessible for integration in larger Python workflows, for example, design involving negative design criteria that complements exact sampling with heuristic optimization (see [28]). Finally, the Infrared implementation can serve as a basis and “rapid prototyping” experimentation platform for future extensions and ideas on multitarget design.

### Sequence alignment

**Fig. 6:**
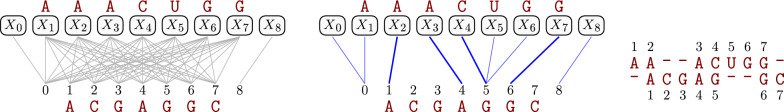
Modeling the sequence alignment of AAACUGG and ACGACGC. From left to right, we illustrate the alignment model $\mathcal {N} _{\text {ali}}$; a valid assignment; the corresponding alignment

Expressing sequence alignment, one of the most prominent problems of bioinformatics, in our framework enables solving various more expressive, even highly complex types of alignment by extending the model. To give an example, we later (Sec. “From sequence alignment to pseudoknot sequence-structure alignment”) discuss the extension to pseudoknotted RNA structure alignment, close to LicoRNA [3]. We start by modeling the elementary problem, which has well-known efficient solutions [42, 43] by classic dynamic programming. The extension of this first model from linear to affine gap cost is discussed in Section “Sequence alignment with affine gap cost”.

#### Definition 13

[Sequence alignment] A **sequence alignment** $\mathcal {A}$ of two **sequences** $a$ and $b$ (both are words over $\Sigma$) is a sequence of pairs (aka **alignment columns**) composed of $(\Sigma \cup \{-\})^2{\setminus } \{(-,-)\}$ such that removing − from the words composed of the first (resp. second) letter of all pairs yields $a$ (resp. $b$). Let $(i,j)$ be a pair in the alignment. We say $(i,j)$ is a match if $i$ equals to $j$, an insertion if $i$ is -, a deletion if $j$ is -, and a mismatch otherwise.

For simplicity, we begin our discussion with **linear gap cost** scoring models, where the score of an alignment $\mathcal {A}$ is defined by gap cost $\gamma$ and an **elementwise score** $\sigma :\Sigma ^2 \rightarrow \mathbb {R}$, as

$$\begin{aligned} {\text {score}} {(\mathcal {A})}=\sum _{i: A_i \,\text {match}} \sigma (A_i) +\# {\text {gaps}}(\mathcal {A}) \gamma , \end{aligned}$$

where ${\# {\text {gaps}}(\mathcal {A})}$ denotes the number of insertions and deletions in $\mathcal {A}$.

Consider two RNA sequences AAACUGG and ACGACGC ( $\Sigma =\{\texttt {A},\texttt {C},\texttt {G},\texttt {U} \}$ ). Assuming similarity scores 2 for matching, and uniformly $-1$ for insertion and deletion, their alignment

$$\begin{aligned} \texttt {A} _1 \texttt {A} _2 - - \texttt {A} _3 \texttt {C} _4 \texttt {U} _5 \texttt {G} _6 \texttt {G} _7 -\\ - \texttt {A} _1 \texttt {C} _2 \texttt {G} _3 \texttt {A} _4 \texttt {G} _5 - - \texttt {G} _6 \texttt {C} _7 \end{aligned}$$

has a score of $6-3-3=0$ due to three matches, three insertions, and three deletions.

The **alignment problem** takes two sequences, denoted $a$ of length *n* and $b$ of length *m*, and an elementwise score $\sigma$. Assuming that $\sigma$ defines a similarity, it asks for maximizing the ${\text {score}} (\mathcal {A})$ over all alignments and an optimal alignment $\mathcal {A}^*$.

*Modeling alignment* We model this problem by introducing one variable $X_i$ per position *i* of the first sequence, whose values indicate their alignment to positions in the second sequence. Before stating our model, we need to resolve a significant issue with this idea. If we express assignments (match/mismatch) between positions *i* of $a$ and *j* of $b$ directly as assignment of *j* to $X_i$ ($x_i=j$) then how do we express deletions of *i*? Naively introducing a *special* value for deletion, e.g. $\bot :=m+1$, makes it *difficult* to express the **noncrossing condition** on assignments, namely the positions *j* of $b$ can be assigned to positions *i* of $a$ in increasing order ($i>i'$ implies $j>j'$). More precisely, naive encoding introduces inequality-like constraints between all pairs of variables $X_i$ and $X_{i'}$ ($1\le i<i'\le n$).

Instead, following [3, 20], we model the deletion of a position *i* by assigning the same value to $X_i$ and $X_{i-1}$. This keeps the assigned values in increasing order and allows a unique representation of alignments by assignments. To further facilitate modeling, we introduce *sentinel* variables $X_0=0$ and $X_{n+1}=m+1$. As illustrated in Fig. 6, our example alignment is then encoded by the assignment

$$\begin{aligned} \begin{array}{c|ccccccccc} i &{} 0 &{} 1 &{} 2 &{} 3 &{} 4 &{} 5 &{} 6 &{} 7 &{} 8 \\ \hline x_i &{} 0 &{} 0 &{} 1 &{} 4 &{} 5 &{} 5 &{} 5 &{} 6 &{} 8 \end{array}. \end{aligned}$$

With this idea (illustrated in Fig. 6), the feature network $\mathcal {N} _{\text {ali}}$ is formalized by

- $$\mathcal {X} _{\text {ali}}=\{X_0,\ldots , X_{n+1}\}$$;
- $$\mathcal {D} _{\text {ali}}=\{0\}\times \{0,\ldots ,m\}^n \times \{m+1\}$$;
- $$\mathcal {C} _{\text {ali}}=\{{\text {\textsf{Leq}}}_{[X_{i-1},X_i]} \mid i\in [2, n]\}$$;
- $$\mathcal {F} _{\text {ali}}=\{F_{\text {match}}, F_{\text {insertion}}, F_{\text {deletion}}\}$$ with
  - $$F_{\text {match}}=\{{\text {\textsf{Match}}}_{[X_i]}\mid i\in [1,n]\}$$;
  - $$F_{\text {deletion}}=\{{\text {\textsf{Deletion}}}_{[X_{i-1},X_i]}\mid i\in [1,n]\}$$.
  - $$F_{\text {insertion}}=\{{\text {\textsf{Insertion}}}_{[X_{i-1},X_i]}\mid i\in [1,n+1]\}$$;

The constraint ${\text {\textsf{Leq}}}_{[X_{i-1}, X_i]}:(x_{i-1}, x_i)\mapsto (x_{i-1}\le x_i)$ ensures an increasing order of the values in the assignment. The network functions express the alignment score:

$$\begin{aligned}&{\text {\textsf{Match}}}_{[X_{i-1},X_i]}(x_{i-1},x_i) \\ {}&\quad = {\left\{ \begin{array}{ll} \sigma (a [i],b [x_i]) &{} x_{i-1}<x_i \\ 0 &{} \text {otherwise} \end{array}\right. } \\&{\text {\textsf{Deletion}}}_{[X_{i-1},X_i]}(x_{i-1},x_i) \\ {}&\quad = {\left\{ \begin{array}{ll} \gamma &{} x_{i-1}=x_i \\ 0 &{} \text {otherwise} \end{array}\right. } \\&{\text {\textsf{Insertion}}}_{[X_{i-1},X_i]}(x_{i-1}, x_i) \\ {}&\quad = {\left\{ \begin{array}{ll} \gamma (x_i - x_{i-1} -1) &{} x_{i-1}\ne x_i \\ 0 &{} \text {otherwise.} \end{array}\right. } \end{aligned}$$

***Efficient solving*** Infrared ’s general solving mechanism computes alignments based on this model in $\mathcal {O}(n \cdot m^2)$ time, dominating the $\mathcal {O}(nm)$ time for the traceback, and $\mathcal {O}(nm)$ space following Propositions 4 and 5 (treewidth 1; *n* variables with domains of size *m*; $\mathcal {O}(n)$ functions, each evaluated in constant time).

Note that while this automatic solution is efficient, it is still more costly than the known dynamic programming alignment algorithms by a linear factor. (In more detail, it does not profit from the linear cost of insertion; one could, within the same complexity, encode nonlinear insertion cost by modifying the functions ${\text {\textsf{Insertion}}}$.) This issue has been discussed and solved before for the case of sequence alignment based on the presented model [3, 20]; essentially it can be solved by applying DP to process single bags. Resolving this issue in broader generality is an open problem, whereas in principle the known specific solutions for sequence alignment can be implemented in the framework.

In practice, this issue is strongly alleviated by *banding strategies* [44] that limit the domain size to $\mu<\!<m$; this reduces the complexity to $\mathcal {O}(n \mu ^2)$ time (and $\mathcal {O}(n \mu )$ space).

## Model extensions and advanced topics

### Sequence alignment with affine gap cost

For more realistic alignments, the cost of consecutive runs of insertions and deletions (aka **gaps**) is scored in a nonlinear fashion; e.g. *k* consecutive insertions are evolutionarily *less costly* than *k* independent insertions. This motivates redefining the score of an alignment $\mathcal {A}$:

$$\begin{aligned} {\text {score}} '(\mathcal {A})=\sum _{i: A_i \,\text {match}} \sigma (A_i) + {\text {gapcost}}(\mathcal {A}), \end{aligned}$$

where generally ${\text {gapcost}}(\mathcal {A}) = \sum _{\text {gap of length}\, \ell \,\text {in}\, \mathcal {A}} g(\ell ).$ For $g(\ell ):=\gamma \ell$, this score degenerates to the case of linear gap cost. The most prominent case is **affine gap cost**, where $g(\ell ):=\beta + \gamma \ell$, distinguishing gap opening $\beta$ from gap extension cost $\gamma$.

Underlining the asymmetry of $\mathcal {N} _\text {ali}$, we could extend the model to arbitrary cost of insertions by redefining ${\text {\textsf{Insertion}}}_{[X_{i-1},X_i]}(x_{i-1},x_i):= g(x_i-x_{i-1}-1)$; however, modeling affine cost for deletions cannot be expressed in a direct modification of ${\text {\textsf{Deletion}}}_{[X_{i-1},X_i]}(x_{i-1},x_i)$ since we lack information to distinguish gap opening and extension.

One can envision at least two possible fixes. First, we can replace the binary deletion network functions with ternary functions that depend on $X_{i-2},X_{i-1},X_i$. This extension comes at the price of increasing the tree width by 1 (and thus the complexity by a further factor of *m*.) Second, we can introduce additional Boolean variables $Y_i$ to reflect the matching state at position *i*: $Y_i$ is assigned to $y_i=1$ if *i* is matched; $y_i=0$, if *i* is deleted. In turn, the deletion function can be modified to depend on $X_{i-1},$ $X_i$ and $Y_{i-1}$:

$$\begin{aligned} & {\text{Deletion}}_{{\left[ {X_{{i - 1}} ,X_{i} ,Y_{{i - 1}} } \right]}} \,(x_{{i - 1}} ,x_{i} ,y_{{i - 1}} )\, \\ &\quad= \,\left\{ {\begin{array}{*{20}l} {\beta + \gamma } \hfill & {x_{{i - 1}} = x_{i} \,{\text{and}}\,y_{{i - 1}} = 0} \hfill \\ \beta \hfill & {x_{{i - 1}} = x_{i} \,{\text{and}}\,y_{{i - 1}} = 1} \hfill \\ 0 \hfill & {{\text{otherwise}}{\text{.}}} \hfill \\ \end{array} } \right. \end{aligned}$$

***Complexity*** For the first idea, we derive a time complexity of $\mathcal {O}(nm^3)$ (Prop. 4), since the treewidth is 2. In the second model, adding Boolean variables (and ternary constraints to relate them to the *X* variables) technically increases the treewidth, but since the variables $Y_i$ have a domain size of 2, in contrast to the linear domain size of the variables $X_i$, their effect on the complexity is much lower (in this case, even constant in sequence length).

Here, the direct application of Proposition 4 would strongly overestimate; instead we follow the argumentation of Section “Complexity analysis for nonuniform domain sizes”. The introduced $Y_i$ variables each correspond to the $X_i$ variable of the same index. Collapsing the nodes of these corresponding variables in the dependency graph, let us us decompose it with width 1. Thus, we bound the time complexity by $\mathcal {O}(n \cdot m^2 2^2)$; see also our discussion of the linear case. The *Y* variables thus contribute a constant factor of 4, comparable to the overhead of Gotoh’s algorithm [43] over linear gap cost alignment (approximately factor 3). Thus, the second model improves the first one by a linear factor—intuitively, it allows sharing Boolean variables between bags instead of variables of linear domains.

### From sequence alignment to pseudoknot sequence-structure alignment

**Fig. 7:**
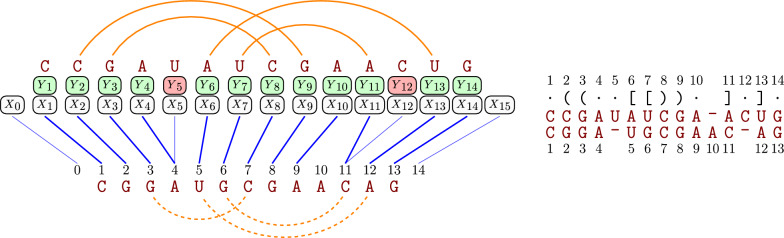
Modeling sequence structure alignment. Example of a valid assignment and corresponding alignment with a pseudoknotted structure. The model contains one network function ${\text {\textsf{BPMatch}}}$ per input base pair (arcs on top). These functions contribute $\tau$ for matches to canonical bases (dashed arcs)

We will develop Infrared models for RNA alignment, where the first RNA is annotated by a potentially crossing secondary structure. We build on the previously described sequence alignment model $\mathcal {N} _{ali}$. Recall our definition of RNA secondary structure from Section “RNA design”; here, we will explicitly consider *general* secondary structures, where base pairs can cross and thereby form arbitrary pseudoknots. This means that we are solving the essentially same optimization problem as LicoRNA [3]. While LicoRNA implements hand-crafted, specialized dynamic programming algorithms, Infrared automatically derives closely related algorithms from a network model, typically from less than 100 lines of Python code. These algorithms solve the pseudoknotted RNA alignment problem efficiently for the same fixed treewidth parameter.

Given are two RNA sequences *a* and *b* of respective length *n* and *m*, additionally a general (i.e. not necessarily noncrossing, potentially pseudoknotted) RNA secondary structure *S* of length *n*; *S* is also called **arc-annotation** of $a$.

We are interested in optimizing a type of alignment score that takes the structural relations due to the arc annotation into account; see Fig. 7. To demonstrate the principle, we extend the sequence alignment score of the previous section by an **arc match bonus** $\tau$. Let us thus define our **sequence structure alignment score** by

$$\begin{aligned} & {\text{score}}_{S} ({\mathcal{A}})\, = \,{\text{score}}({\mathcal{A}})\, \\ &\quad+ \,\sum\limits_{{\left( {i,j} \right) \in S}} {\left\{ {\begin{array}{*{20}c} \tau & {{\mathcal{A}}{\text{ matches }}i {\text{ to }}i^{\prime}} \\ {} & {{\text{and }}j{\text{ to }}j^{\prime};(b[i^{\prime}],b[j^{\prime}]) \in {\mathcal{B}}} \\ 0 & {{\text{otherwise}},} \\ \end{array} } \right.} \end{aligned}$$

where $\mathcal {B}$ is the set of canonical base pairs (Sec. “RNA design”).

#### Problem 5

[General sequence-structure alignment] Given sequences *a*, and *b* annotated by *S*, the **sequence structure alignment problem** asks for a sequence alignment of *a* and *b* (Def. 13) that optimizes the sequence structure alignment score ${{\text {score}}_S} (\mathcal {A})$.

Our feature network model $\mathcal {N} _\text {sali}$ directly builds on $\mathcal {N} _\text {ali}$, extending it by network functions to encode the structure component of the score. As discussed in the previous subsection (for the purpose of modeling affine gap cost), we introduce Boolean variables $Y_i$ to indicate the match of position *i* in $a$ since they let us express the arc match bonus more efficiently. We obtain

- $$\mathcal {X}_\text {sali} = \mathcal {X}_\text {ali}\cup \{Y_1,\dots ,Y_n\}$$;
- $\mathcal {D}_\text {sali}$ extends $\mathcal {D}_\text {ali}$ by Boolean domains {0,1} for all $Y_i$;
- $$\mathcal {C}_\text {sali} = \mathcal {C}_\text {ali}\cup \mathcal {C}_\text {relXY}$$;
- $$\mathcal {F}_\text {sali} = \mathcal {F}_\text {ali}\cup \{F_\text {bpmatch}\}$$;

where $\mathcal {C}_\text {relXY}$ is a set of constraints that relate the variables $Y_i$, $X_{i-1}$ and $X_i$, such that $y_i=1 \iff x_{i-1}<x_i$ (for all $1\le i\le n$) and $F_\text {bpmatch}=\{{\text {\textsf{BPMatch}}}_{[X_i,X_j,Y_i,Y_j]} \mid (i,j)\in S\}$

$$\begin{aligned}&{\text {\textsf{BPMatch}}}_{[X_i,X_j,Y_i,Y_j]}(x_i,x_j,y_i,y_j) \\ {}&\quad = {\left\{ \begin{array}{ll} \tau &{} y_i=1, y_j=1, \text { and } (b[x_i],b[x_j]) \in \mathcal {B} \\ 0 &{} \text {otherwise}. \end{array}\right. } \end{aligned}$$

Note that ${\text {\textsf{BPMatch}}}$ (for an arc $(i,j)\in S$) cannot be defined in dependency of only $X_i$ and $X_j$, since $(b[x_i],b[x_j]) \in \mathcal {B}$ could hold in cases where *i* or *j* are deleted.

***Complexity*** As in the analysis of Section “Sequence alignment with affine gap cost”, we collapse each pair of nodes of variables $X_i$ and $Y_i$ (of the same index *i*) in the dependency graph. The result is isomorphic to the *structure graph* of RNA *a*, consisting of its nucleotides as nodes, and edges due to its backbone and base pairs. For the treewidth $w$ of this graph, we derive $\mathcal {O}(n2^{w +1}m^{w +1})$ time complexity by Corollary 1.

Whereas in our models for network parsimony or RNA design the domain size is constantly bounded, here it depends on the input size. Consequently, solving of this RNA alignment problem is not in parameterized complexity class **FPT**, but **XP** (Sec. “Parameterized complexity classes”).

***Discussion*** The presented model extension yields an automatically derived solution to the pseudoknot sequence-structure alignment problem with parameterized complexity in the treewidth. Compared to LicoRNA, our algorithms depend on the exact same fixed parameter. Note that, in the current implementation, Infrared’s complexity is worse by a linear factor due to the same reason as we discussed for sequence alignment before. In practice, this is often reduced to a constant factor, namely the band width.

This is contrasted by general benefits due to the declarative implementation in Infrared (Jupyter notebook). For example, the code is well maintainable, extensible by further constraints and evaluation criteria, and can profit from future developments and optimization of the Infrared system.

### Finite state automata

**Fig. 8:**
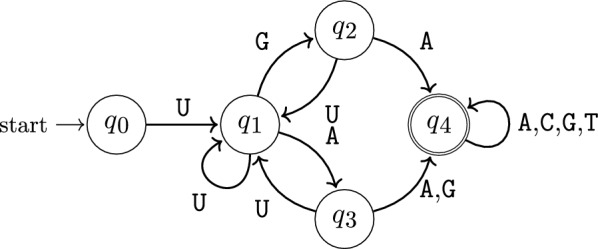
Sketch of the 5-state Deterministic finite “Aho-Corasick” automaton accepting the three stop codons UGA, UUA, UUG. We do not draw back-transitions to $q_0$, which occur implicitly for all not explicitly shown cases (i.e.  A,C,G in $q_0$; C in $q_1$, C,G in $q_2$; and C in $q_3$). To *forbid*, instead of accept, all of the three stop codons, we complement the language by making all states but $q_4$ accepting

A common side condition when designing RNA or DNA sequences is to avoid or enforce certain sequence motifs. For example, one could be interested in avoiding stop codons anywhere in the designed sequence (or avoid restriction sites, enforce binding sites...). Such conditions can be generalized in terms of regular languages, accepted by deterministic finite state automata (DFA; Fig. 8). This idea is well known in constraint programming; for example it is the basis of the global regular language membership constraint [45]. In the specific case of sequence design, DFAs have been introduced by [46] to perform such tasks efficiently for a set of sequence motifs. We show that finite state automata can be emulated in network models. Remarkably, this allows us to efficiently handle such requirements even in combination with other design objectives; e.g. the automaton model of this section could be merged with our model for multitarget RNA design $\mathcal {N} _{\text {design}}$ (Sec. “RNA design”).

The model is a good example for the use of several types of variables, as we are going to introduce, for every sequence position, one variable to model the nucleotide and one to model the automaton state.

#### Definition 14

[Deterministic Finite Automaton] A **Deterministic Finite Automaton (DFA)** is a $5$-tuple $(\mathcal {Q},\Sigma ,\delta ,q_0,\mathcal {Q}_F)$ with

- Finite set of states $\mathcal {Q}$;
- Finite set of symbols $\Sigma$;
- Transition function $$\delta :\mathcal {Q}\times \Sigma \rightarrow \mathcal {Q}$$;
- Initial state $$q_0\in \mathcal {Q}$$;
- Set of final, accepting states $\mathcal {Q}_F\subset \mathcal {Q}$.

A word $w=a \ldots w_n$ of length $n$ is accepted by a DFA if there exists a sequence of states $q=\{q_0,\ldots , q_n\}\subset \mathcal {Q}^{n+1}$ starting with initial state $q_0$ such that $q_n\in \mathcal {Q}_F$ and $\delta (q_{i-1}, w_i)=q_i$ for all $i\in [1,n]$.

By modeling a DFA as a network model, we can use Infrared to sample accepted words. We consider two types of variables, one for the word $w$ and the other for the state sequence $q$. Given a DFA, the accepted word sampling problem is formalized by the feature network $\mathcal {N} _{\text {DFA}}$ as follows:

- $$\mathcal {X} _\text {DFA}=\{X_1,\ldots ,X_n\}\cup \{Y_0,\ldots ,Y_n\}$$;
- $$\mathcal {D} _\text {DFA}=\Sigma ^n \times \{q_0\} \times \mathcal {Q}^{n-1}\times \mathcal {Q}_F$$;
- $$\mathcal {C} _\text {DFA}=\{{\text {\textsf{Transition}}}_{[X_i,Y_{i-1},Y_i]} \mid i\in [1,n]\}$$;
- $$\mathcal {F} _\text {DFA}=\{\}$$.

The constraint ${\text {\textsf{Transition}}}_{[X_i, Y_{i-1}, Y_i]}:(x_i,y_{i-1},y_i)\mapsto (y_i=\delta (y_{i-1},x_i))$ encodes the DFA transition function. This ensures that, in each sampled assignment, $y_0\ldots y_n$ is the state sequence of the word $x_1\ldots x_n$, which is accepted by DFA as the domain of $Y_n$ is the set of final states $\mathcal {Q}_F$.

***Complexity*** Again, we collapse the variables $X_i$ and $Y_i$ for the same *i* in the dependency graph; let $w$ be the treewidth of the collapsed graph. We obtain time complexity $\mathcal {O}(4^{w +1}|\mathcal {Q}|^{w +1} n)$ by Cor. 1, where $w =1$ for the pure automaton model (without extensions).

When forbidding/enforcing motifs in other design settings, e.g. single or multitarget RNA design, the treewidth typically increases since the automaton model causes dependencies between variables of consecutive positions *i* and $i+1$, while e.g. RNA design defines dependencies between nonconsecutive positions *i* and *j* for each target base pair (*i*, *j*). Based on this analysis, we achieve efficiency equivalent to that of the hand-crafted algorithm [46]. Since the domain size depends on the input size, specifically the number of states, this is another example of solving by Infrared in **XP** (Sec. “Parameterized complexity classes”).

The complexity due to the automaton should be compared to simpler ideas to enforce/forbid motifs of maximum size *k*. More naively, one could introduce such requirements by *k*-ary constraints on each run of *k* consecutive variables $X_{i},\dots ,X_{i+k-1}$ (for all $1\le i\le n-k+1$). This idea results in $\mathcal {O}(4^k n)$ without additional constraints. Automata thus offer a favorable trade-off between domain size and treewidth/exponent (as advocated in [46]). Infrared supports adapting the strategy to the concrete problem.

### Multidimensional Boltzmann sampling

**Fig. 9:**
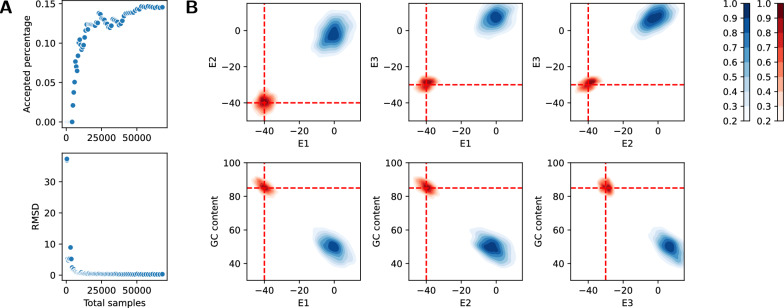
Multidimensional Boltzmann sampling applied to RNA design. For the example of Fig. 5, we target G C content 85% and respective energies E1=-40, E2=-40, E3=-30 for the target structures (with tolerances of 5% GC content and 0.5 kcal/mol energy). Infrared ’s multidimensional Boltzmann sampling (MDBS) strategy starts from uniform sampling (weights 0 for every feature). It iteratively generates Boltzmann samples and updates the weights to move the (estimated) expectation closer to the targets. A Accepted samples as well as root mean square distance (RMSD) to the targets during this procedure, which considered over 70,000 total samples to generate 100 targeted samples. B Kernel density estimate plots: distributions of features for uniform sampling (blue) and sampling at the end of the MDBS run (red), where distributions are shifted to the targets (dashed red lines)

**Algorithm 4:**
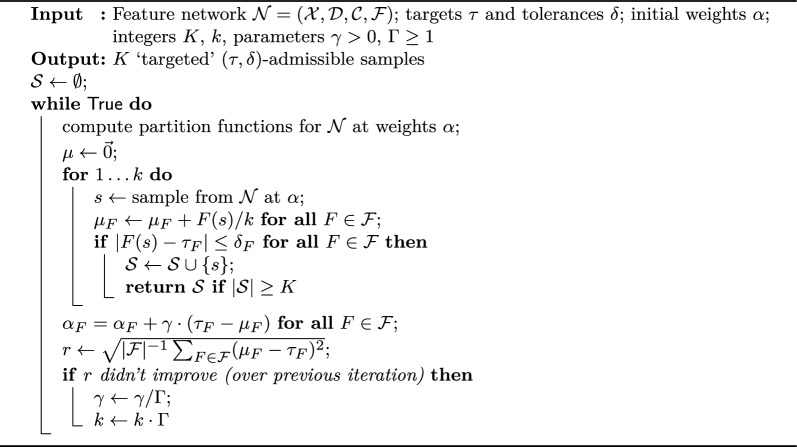
Multidim. Boltzmann sampling

Recall that Section “RNA design” demonstrated random generation of solutions by sampling from the Boltzmann distribution defined by multiple features and weights. The histograms from Fig. 5 show the feature distributions resulting from large negative weights for all features in a multitarget RNA design example. In the example, this allows us to produce designs with low target structure energies and G C content.

Substantially extending the level of control, Infrared supports the random generation of objects with narrowly defined target feature values based on multidimensional Boltzmann sampling (MDBS) [15]. This technique was successfully demonstrated before in RNA design: generating sequences with defined dinucleotide frequencies [47], targeting G C content in single target RNA design by IncaRNAtion [48] and generating RNA designs with specific energies of multiple structures and specific G C content by RNARedprint [16].

*Problem statement* Concretely, given a network $\mathcal {N} =(\mathcal {X},\mathcal {D},\mathcal {C},\mathcal {F})$, we look at the problem of randomly generating valid assignments *x* that satisfy constraints

$$\begin{aligned} |F(x)-\tau _F| \le \delta _F, \end{aligned}$$

for given target values $\tau _F$ and tolerances $\delta _F$ for all (or a subset) of the features $F\in \mathcal {F}$. Let us call such assignments $(\tau ,\delta )$ **-admissible**.

*MDBS strategy and algorithm* As shown in [15] the problem can be solved effectively, under certain assumption even with provable efficiency, by MDBS. This strategy combines rejection sampling, which accepts only $(\tau ,\delta )$-admissible samples, with a learning strategy to maximize its efficiency.

Here, we observe that rejection sampling is most effective, when the targeted values $\tau _F$ coincide with the means of the sampled distributions; moreover, these means depend on the feature weights $\alpha$.

Therefore, Infrared ’s MDBS algorithm (Algorithm 4), starting from initial weights $\alpha$ (by default, $\alpha =\textbf{0}$), iteratively generates *k*-many samples per round. In every iteration, it tweaks the weights $\alpha$ aiming to shift the sampling means closer to the targets; the update step-size is controlled by the tweaking factor $\gamma$. The procedure is repeated until *K*-many $(\tau ,\delta )$-admissible samples are generated. To stabilize this heuristic strategy, Infrared additionally implements an annealing scheme based on improvement of the root mean square deviation (RMSD) to the targets and controlled by the cooling factor $\Gamma$.

*MDBS for RNA design* Fig. 9 illustrates this MDBS strategy for the example of Fig. 5 and specific energy and G C content targets. Showing typical behavior, the strategy improves the RMSD while generating admissible and nonadmissible samples. In this way, it increases the efficiency of generating admissible assignments (Fig. 9A). Figure 9B shows how MDBS shifts the multivariate distribution toward the targets (here starting from uniform sampling).

*Targeting by proxy* The multitarget design example showcases an interesting extension of the standard MDBS mechanism. Namely, in this case, we distinguish base pair energy from Turner energy. To target the latter, we use base pair energies as proxies, since they allow much more efficient sampling (and are sufficiently correlated to Turner energies; compare [16]). To shift the distributions during the MDBS algorithm, we thus estimate the means of the *Turner energies*; then, based on their difference from the target Turner energies, we update the weights of the corresponding *base pair energy* feature. Our Infrared implementation supports ‘targeting by proxy’ in a generalized way (using a second kind of feature *F* whose evaluations *F*(*x*) are defined explicitly, instead of being induced by their network functions).

*Available code examples* In supplemental online material (Jupyter notebooks), we show the code to produce the samples and plots for Fig. 9; as a further example, we demonstrate effective random sequence generation targeting all 16 dinucleotide frequencies of a SAM riboswitch (RF00162; from *S. thermophilum*), while maintaining compatibility with its pseudoknotted RNA structure.

## Implementation

**Fig. 10:**
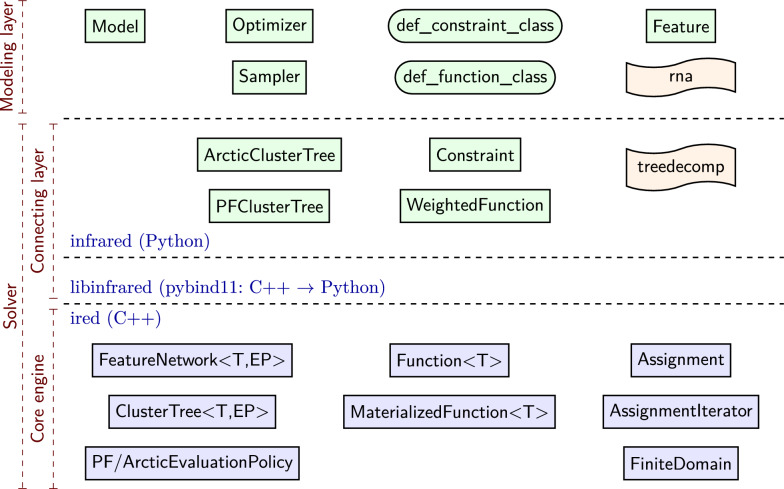
Overview of the Infrared architecture. The C++ **core engine** is connected to a high-end **modeling layer** in Python by a hybrid **connecting layer**. The C++ core implements the computational engine to evaluate forward and traceback algorithms on cluster trees as generic code (e.g. supporting different algebras as evaluation policies; PF for partition function, Arctic for maximization). This optimized low-level layer is exposed to Python using pybind11; the core functionality is moreover extended (by tree decomposition, using module treedecomp, population of the cluster tree...) on the Python side to implement the full computational engine. Finally, the high-level modeling interface of Infrared offers functionality to model and solve feature networks, perform sampling targeting specific features (multidimensional Boltzmann sampling), define custom constrain and function types. Moreover, we include the module rna for RNA-specific functionality

The Infrared software supports declarative modeling of problems as feature networks and treewidth-dependent efficient solving through a high-level Python interface. Figure 10 sketches its architecture. For solving, the software relies on optimized implementations of the presented algorithms in C++. The low-level C++ engine is glued to a high-level ‘modeling’ layer by a pybind11-based C++/Python interface. Thus, Infrared maintains a small algorithmic core in C++ (for high performance), while extending functionality in Python (for increased flexibility). For example, the C++ **core engine** solves cluster trees and focuses on *necessary* functionality, while the construction of the cluster tree from a model, as well as “high-level” functionality such as the declarative composition of models, are implemented in Python.

The C++ code is templated to generically support different function types and evaluation algebras, which keeps the code compact and maintainable; moreover it prepares future extensions of the system. For fast processing of bags, the core engine relies on fast backtracking enumeration of partial assignments (class AssignmentIterator), where constraints and functions are evaluated as early as possible (to avoid unnecessary and redundant computation). Bag processing evaluates constraints ($\texttt {Function<bool>}$) and functions ($\texttt {Function<double>}$) and, in the forward phase, computes messages, stored in memory as objects of $\texttt {MaterializedFunction<double>}$.

For completion of the Infrared solver, the **connecting layer** exposes the C++ functionality to Python, specializing the templates to optimization (arctic policy) and partition function over real-valued features. Moreover, it extends the core by Python wrapper classes that ‘know’ how to construct cluster trees from feature networks. To prepare the definition of function and constraint classes, it wraps the Boolean and real-valued C++ function classes. Generating tree decompositions is delegated to the module treedecomp. While we provide interfaces to different external tree decomposers and support customization, the implemented default strategy applies a randomized min-fill-in heuristic [13] and returns a tree decomposition with heuristically minimized tree width.

Finally, the **modeling interface** layer enables a declarative style of defining feature networks as objects of the class Model through Python code. Adding variables, constraints, and functions supports naturally specifying, but also extending and merging feature networks. From models one can construct solvers to perform different tasks, including optimization, Boltzmann sampling at specified feature weights or targeted sampling; the latter relies on a multidimensional Boltzmann strategy that learns weights to effectively target specific feature values.

*Definition of application-specific constraints and functions* Infrared supports the definition of constraints and functions in Python by special concise syntax via the respective functions def_constraint_class and def_function_class. Examples of their use were given before for defining the constraint NotEquals and network function Card for the introductory graph coloring model. To create Python classes of constraints or network functions, the user calls these functions with the class name and two Python functions. The first function (init) has two roles. It defines the arguments of the constructor and returns the scope (or dependency list) of the constraint or function. The second function (value) defines how the constraint/function is evaluated at specific values of the dependency variables. Using arguments of the same name, information can be passed from initialization to evaluation; e.g. this enables constraint/function type arguments or auxiliary data structures. For clear semantics (while allowing optimizations), we require referential transparency, i.e. the result of the value function must not vary with anything but its arguments.

*Precomputation* The core engine precomputes constraints and network functions when they are added to the cluster tree. For this purpose, they are evaluated for all partial assignments and the results are tabulated (MaterializedFunction). This simple mechanism supports the convenient specification of constraints and functions in Python, while resulting in fast computation times in practice (by significantly reducing the overhead due to the Python computation). From a theoretical perspective, this mechanism preserves the worst case time complexity, since *k*-ary constraints and functions impose a bound on the treewitdh $w \ge k-1$. The strategy requires additional space in $\mathcal {O}(d ^k\mathfrak {m})$, for maximum domain size $d$ and $\mathfrak {m}$ constraints and functions. Space and time consumption due to the precomputation are thus dominated by the solving complexity if $w > k-1$. In this way, the strategy is optimized for the typical performance-critical cases. Future implementations can speed up the precomputation by possibly lazy caching mechanisms without changing semantics and the interface.

## Discussion

The Infrared framework was motivated by the success of related technology in solving complex bioinformatics problems; most directly, by our work on multitarget RNA design [16]. Thus, the system started out as a library that generalized the fixed-parameter tractable (FPT) sampling algorithm of RNARedprint and its multidimensional Boltzmann sampling strategy. Since then it has been developed into a broadly applicable framework, supporting convenient declarative modeling of problems with multiple features, where models can be solved by a generic treewidth-based algorithm using different algebras. This text was written to supply the reader with a comprehensive discussion of the techniques combined in the Infrared system. In contrast, our book chapter [28] focused on coding of design problems in Infrared and did not in-depth discuss Infrared’s methods in favor of an application-oriented introduction. Furthermore, note that we published a book chapter on the first version of RNARedprint [49], which did not make use of the Infrared system.

Since the system’s first application for concisely reimplementing RNARedprint with improved functionality and performance, it has proven to be a very useful tool for further algorithmic developments in bioinformatics [17, 19, 50]. Other previous work [3, 20, 46, 48] could have directly profited from the Infrared framework. For several previous algorithms [3, 17, 46, 48], we presented feature network models and discussed their solving complexity. For these examples, the system yields essentially identical algorithms; with the exception of alignment, where Infrared lacks a problem-specific optimization (Secs. “Sequence alignment” and “From sequence alignment to pseudoknot sequence-structure alignment”), which we plan to add in the future. From the well-researched field of network parsimony, we present further examples where the Infrared ’s solving complexity is on par with state-of-the-art algorithms [5] (even improving softwired parsimony on binary networks).

*Utiltity for prototyping and practical applications* In summary, these previous works witness the suitability of Infrared for prototyping novel algorithmic ideas; moreover, their benchmark results show the practical utility of the system to solve relevant problem instances. Of particular interest, we show that—for many practically relevant problem instances—the treewidth is sufficiently low to enable effective solving by Infrared.

**Fig. 11:**
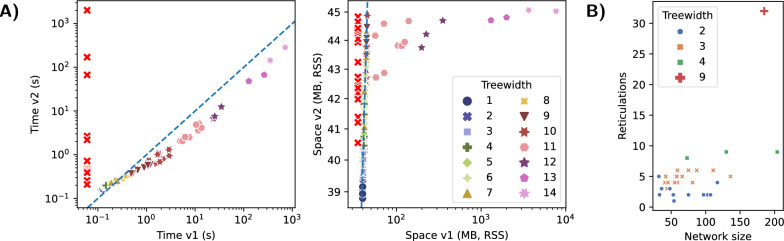
**A** Benchmark comparison of the Infrared-based RNARedPrint v2 to the original C++ implementation “v1”. Time is measured as user time; space, as maximum resident set size (RSS). We run the tools on the RNAfold benchmark set [16]. We let both tools generate 1000 samples at fixed weights; note that time and space are strongly dominated by the precomputation phase. To directly compare the implementations of the core algorithms, we run both tools on identical tree decompositions, although Infrared ’s default tree decomposer improves for several instances (including the most expensive one). One observes that the RNARedPrint v2 improves in space and time over the original implementation. Only for very short runtimes, at low treewidth, the C++ implementation has a slight edge, presumably due to less overhead. Both implementation show almost no noticable space increase at low tree widths; however the space requirements of the original implementation increase dramatically for treewidths larger than 10. Due to its extreme space requirement, we didn’t solve the single instance of treewidth 16 with RNARedPrint v1; in other cases, it failed due to a bug. For those instances, we indicate only the performance of version 2 (red crosses). **B** We used Infrared to compute the treewidths for a set of various phylogenetic networks that were collected from recent studies [52]. Using the Infrared network parsimony model, we count the number of reticulation nodes in the networks and calculate their treewidth. It can be seen that the treewidth rather correlates with the number of reticulation nodes than with network size (number of nodes and edges). Our study on ’real-world’ phylogenetic networks suggests that treewidths are often low in practice; consequently Infrared can effectively compute network parsimony by solving the presented models

In addition, we wanted to learn about Infrared ’s practical performance in relation to optimized problem-specific code in a high-performance computing language (Fig. 11A). Taking a unique opportunity, we compared our original C++ implementation of multitarget design [16, 49], RNARedprint  v1, to our Infrared-based reimplementation RNARedprint  v2. We chose the benchmark set “RNAfold”, e.g. used in [16, 51], comprising 400 design instances of 3–6 structures, which were generated to pose ambitious challenges with treewidths up to 16. This experiment was performed on an Intel Core i7-4770 CPU with 32 GB memory.

Moreover, we studied the practical applicability of treewidth-based network parsimony algorithms (Sec. “Network parsimony”) on a set of nonartificial phylogenetic networks [52] compiled from the literature (see https://phylnet.univ-mlv.fr/recophync/networkDraw.php). This data set is typically used as a reference set for the comparison and evaluation of various algorithms on phylogenetic networks. For our purposes, we determined the treewidths of the networks in dependency on their size and the number of reticulation nodes (Fig. 11B). Here treewidth directly provides information about the solving efficiency of hardwired and softwired parsimony problems (Sec. “Network parsimony”). The low to moderate treewidths on these instances hint at permissible performance in many real-word scenarios.

*Characteristics and application range* By modeling a series of concrete bioinformatics problems, we showed that Infrared is broadly applicable, going well beyond the selected examples. As discussed before, this extends to applicability *in practice*, where Infrared can efficiently solve relevant instances of expressible problems. Although feature networks are virtually universal, such that they do not limit the system’s expressivity, Infrared ’s solver relies on a very specific mechanism, where efficiency strictly depends on the treewidth of the problem instance. Arguably, this is a prerequisite for the very characteristic properties of the framework. In contrast to heuristic methods, our tree decomposition-based solving strategy leads to predictable worst-case complexity guarantees for exact optimization *and* sampling. Notably, exact controlled sampling rules out many heuristic pruning-type solving strategies, since it requires exact computation of partition functions.

Nevertheless, the dependency on treewidth necessarily limits the *practically solvable* problems and instances. In practice, such problems (explicitly or implicitly) have some graph structure. Examples are graph coloring or multitarget RNA design, which both are NP hard, but efficiently solvable for specific instances, whose graphs are sufficiently close to trees.

Infrared was designed to handle such tree decomposable problems (and their low-treewidth instances) well, but its general solving mechanism offers the flexibility to go beyond. For example, the framework supports strategies that limit the treewidth of considered instances (e.g. we controlled the maximum treewidth in our negative design approach RNAPOND [17]) or reduce the treewidth in controlled ways (e.g. TreeDiet [18]). For this class of problems, we identify potential for future improvements of the Infrared solver, which could allow instances to be solved significantly faster or with better complexity. For example, we discussed the complexity of alignment; additionally, consistency-based methods can yield significant improvements over the current evaluation strategy by cluster tree elimination.

*Delimitation* The system is, however, not designed and is even unsuitable for problems that cannot be modeled as a decomposable feature network. This comprises many constraint satisfaction (CSP) or constraint optimization problems (COP) that would typically be solved by general constraint solvers (e.g. the constraint programming system Gecode [26]), SAT solving (e.g. MiniSAT [53]), or solving of integer linear programs (e.g. CPLEX [54]). In many classical CSP examples (e.g. n-Queens, Sudoku), models induce a complete (or almost complete) dependency graph. Here, Infrared does not implement strategies to heuristically cope with the exponential worst case complexity (e.g. constraint propagation or branch and bound); its solving strategy would therefore be essentially brute force. Obviously Infrared is thus not a general solver for CSP/COP, even if its declarative modeling paradigm and interface remind of such systems.

While Infrared proposes a novel form of automated solving of declarative problem specifications by dynamic programming (DP), bioinformatics already has a long tradition of combining declarative methods and dynamic programming in the form of Algebraic Dynamic Programming (ADP) [55].

Despite the common DP backdrop, Infrared and ADP pursue different, even orthogonal goals: instead of deriving a DP algorithm from a declarative problem description, ADP implementations such as GAPC [56] or ADPFusion [57, 58] aim to support the implementation of DP algorithms through algebraic abstraction.

## Conclusions

We presented a framework for rapidly developing applications that make use of efficient exact optimization and sampling techniques by declaratively specifying problems in Python. As such, the framework provides flexible access to recent advanced algorithmic techniques—while specifying problems resembles common constraint modeling systems.

The system allows modeling problems as feature networks, which we introduced as a form of weighted constrained networks that support several features. The main advantages and characteristics of the framework stem from combining expressive modeling with automated combinatorial solving strategies that support exact optimization and weighted sampling. In particular, exact sampling, which requires complete combinatorial algorithms, can be used in innovative ways. For example, it allows generating decoys and background models in complex settings or targeting multiple features in its extension to multidimensional Boltzmann sampling.

As elaborated, these tasks are performed by generic solving algorithms based on tree decompositions of the models. Being parameterized by the treewidth, this strategy profits from the often moderately low treewidth of many typical problems in bioinformatics.

We underline the broad range of possible applications, by our discussion of diverse application examples and their implementation (online documentation). Demonstrating the concise reimplementation of previous bioinformatics methods, these applications serve as reference coding examples and also show the practical relevance of the framework.

Crucially, the system makes such methods accessible through a declarative interface in Python. Since this strongly facilitates their flexible use, the system promotes future applications of these techniques. Increasing flexibility, the system supports extension and refinement of existing models as well as their composition, e.g. sequence design targeting structure RNA *and* forbidding specific sequence motifs.

*Future work* We plan to further optimize the Infrared solver due to consistency methods and/or forward checking (in single bag processing). In specific cases, such techniques even improve complexity bounds over the currently implemented CTE-like evaluation mechanism. Moreover, we want to adapt the linear-factor speedup over standard evaluation for alignment problems (LicoRNA)—generalizations of this technique pose interesting research questions. As another path of optimization, we will implement improved tree decomposition adapted to our solver.

Furthermore, the architecture of the framework enables additional solvers; for example, such solvers can compute Pareto-optima or perform nonredundant sampling [59] based on feature network models. Moreover, it will be interesting to explore the use of further evaluation algebras, which can be used with the generic evaluation algorithms of Infrared ’s C++ core engine.

Finally, the system highlights benefits due to tree decomposition of problems that enable general solving by efficient combinatorial methods. While such methods can be tedious to implement from scratch, we demonstrated their use through a declarative modeling interface. In future work, we envision developing and making related methods accessible in a similar way. One exciting line would extend our work on tree decomposition-based automatic generation of dynamic programming schemes [19].

### Supplementary Information

**Additional file 1. Correctness proofs**. The file contains proofs of Propositions 1, 2 and 3, which establish the correctness of the optimization and sampling algorithms.

## Footnotes

1 https://gitlab.inria.fr/amibio/RNARedPrint.
