# Supplementary material for: Infrared: a declarative tree decomposition-powered framework for bioinformatics
Source: Algorithms Mol Biol. 2024 Mar 16;19:13. doi: 10.1186/s13015-024-00258-2 (PMC10943887; doi:10.1186/s13015-024-00258-2)
Supplement: Supplementary file 1 — Additional file 1. Correctness proofs. The file contains proofs of Propositions 1, 2 and 3, which establish the correctness of the optimization and sampling algorithms. [file 13015_2024_258_MOESM1_ESM.pdf]

Infrared: a declarative tree decomposition-powered framework for bioinformatics

Additional file 1

Hua-Ting Yao, Bertrand Marchand, Sarah J. Berkemer, Yann Ponty, Sebastian Will

Recall the following few definitions and notation from the main text. Given the cluster tree (T, χ, ϕ) and a node u of T. If u has the parent v, then $\mathrm{diff}(u) = \chi(u) \setminus \chi(v)$ and $\mathrm{sep}(u) = \chi(v) \cap \chi(u)$; otherwise, if u is the root, $\mathrm{diff}(u) = \chi(u)$ and $\mathrm{sep}(u) = \emptyset$. Moreover, we defined T_u as the subtree under node u, $\chi(T_u) = \bigcup_{u' \in T_u} \chi(u')$ and $\mathrm{diff}(T_u) = \chi(T_u) \setminus \mathrm{sep}(u)$. We use the following lemma.

Lemma S1. $\{\mathrm{diff}(T_c) \mid c \text{ child of } u\}$ is a partition of $\chi(T_u) \setminus \chi(u) = \mathrm{diff}(T_u) \setminus \mathrm{diff}(u)$.

Proof. Let u be a node of the cluster tree (T, χ, ϕ), c and c' be children of u, then $\mathrm{diff}(T_c) \cap \mathrm{diff}(T_{c'}) = \emptyset$, since—due to tree decomposition property (3)—any common element must be in $\chi(u)$ in contradiction to the definition of diff on subtrees; moverover,

$$\begin{aligned}\bigcup\{\mathrm{diff}(T_c) \mid c \text{ child of } u\} &= \bigcup_{c \text{ child of } u} \chi(c) \setminus \mathrm{sep}(c)\\ &= \bigcup_{c \text{ child of } u} \chi(c) \setminus \bigcup_{c \text{ child of } u} \mathrm{sep}(c)\\ &= \bigcup_{c \text{ child of } u} \chi(c) \setminus \chi(u) = \mathrm{diff}(T_u) \setminus \mathrm{diff}(u).\end{aligned}$$

□

1 Correctness of optimization

Proof of Proposition 1. We are going to show the correctness by structural induction on the cluster tree. For the base case, let u be a leaf of the cluster tree. Sinc the subtree T_u is the node u itself, Eq. 3 for a leaf u is written as

$$m_{u \to v}(\bar{x}) = \max_{\substack{\bar{x}' \in \mathcal{A}_{\mathrm{diff}(u)} \\ \bar{x} \cup \bar{x}' \text{ is valid}}} \sum_{f \in \phi(u)} \alpha_f f(\bar{x} \cup \bar{x}').$$

Thus, Proposition 1 holds for leaves u, since they do not have children.

For the induction step, let u be an internal node of the cluster tree and assume Proposition 1 holds for all children of u. Denote the children of u as $c_1 \dots, c_k$. We directly apply the induction hypothesis to the r.h.s of the claim.

$$\text{r.h.s. } (4) = \max_{\substack{\bar{x}' \in \mathcal{A}_{\mathrm{diff}(u)} \\ \bar{x} \cup \bar{x}' \text{ is valid}}} \left[\begin{array}{l} \sum_{f \in \phi(u)} \alpha_f f(\bar{x} \cup \bar{x}') \\ + \sum_{c_i \text{ child of } u} \max_{\substack{\bar{y}_i \in \mathcal{A}_{\mathrm{diff}(T_{c_i})} \\ \bar{x} \cup \bar{x}' \cup \bar{y}_i \text{ is valid}}} \sum_{f \in \phi(T_{c_i})} \alpha_f f(\bar{x} \cup \bar{x}' \cup \bar{y}_i) \end{array} \right]$$

Our goal is to show equivalence to the r.h.s. of Eq. (3). By Lemma S1 set $\mathrm{diff}(T_u) \setminus \mathrm{diff}(u)$ is disjointly decomposed into $\mathrm{diff}(T_{c_1}) \dots \mathrm{diff}(T_{c_k})$; moreover, $\phi(T_u) \setminus \phi(u)$ is partitioned into $\phi(T_{c_i})$, $i = 1 \dots k$. It follows that for every fixed assignment $\bar{x} \cup \bar{x}'$, the maximizations over $\bar{y}_i \in \mathrm{diff}(T_{c_i})$ are independent for different i; as well the sum over the functions of bag u is independent of the maximizations. Thus, we can move the maximizations over the assignments $\bar{y}_i$ in front of the summations.

$$= \max_{\substack{\bar{x}' \in \mathcal{A}_{\mathrm{diff}(u)} \\ \bar{x} \cup \bar{x}' \text{ is valid}}} \max_{\substack{\bar{y}_1 \in \mathcal{A}_{\mathrm{diff}(T_{c_1})} \\ \bar{x} \cup \bar{x}' \cup \bar{y}_1 \text{ is valid}}} \cdots \max_{\substack{\bar{y}_k \in \mathcal{A}_{\mathrm{diff}(T_{c_k})} \\ \bar{x} \cup \bar{x}' \cup \bar{y}_k \text{ is valid}}} \left[\begin{array}{l} \sum_{f \in \phi(u)} \alpha_f f(\bar{x} \cup \bar{x}') \\ + \sum_{c_i \text{ child of } u} \sum_{f \in \phi(T_{c_i})} \alpha_f f(\bar{x} \cup \bar{x}' \cup \bar{y}_i) \end{array} \right]$$

Then, we combine the maximizations over the single partial assignments $\bar{y}_i$; moreover we replace the sums with a single sum ranging over the combined assignments $\bar{y}$ of $\text{diff}(T_u) \setminus \text{diff}(u)$.

$$= \max_{\substack{\bar{x}' \in \mathcal{A}_{\text{diff}(u)} \\ \bar{x} \cup \bar{x}' \cup \bar{y} \text{ is valid}}} \max_{\substack{\bar{y} \in \mathcal{A}_{\text{diff}(T_u) \setminus \text{diff}(u)} \\ \bar{x} \cup \bar{x}' \cup \bar{y} \text{ is valid}}} \left[\sum_{f \in \phi(T_u)} \alpha_f f(\bar{x} \cup \bar{x}' \cup \bar{y}) \right]$$

The last term equals r.h.s. of Eq. (3), showing the induction step. □

2 Correctness of partition function computation

Proof of Proposition 2. Again, we will show the correctness by induction. Starting with the base case where u is a leaf of the cluster tree, the subtree T_u is the node u and the child set of u is empty. Eq 5 is then written as

$$m_{u \to v}(\bar{x}) = \sum_{\substack{\bar{x}' \in \mathcal{A}_{\text{diff}(u)} \\ \bar{x} \cup \bar{x}' \text{ is valid}}} \prod_{f \in \phi(u)} \exp(\alpha_f f(\bar{x} \cup \bar{x}'))$$

which shows the correctness for leaves.

For the induction step, let u be an internal node of the cluster tree; we assume that the message $m_{c_i \to u}$ for all children c_i, $i \in \{1, \ldots, k\}$ of u is correctly computed as in Proposition 2. Following the scheme as in the previous proof, we first rewrite the r.h.s of the claim applying the induction hypothesis,

$$\text{r.h.s. (6)} = \sum_{\substack{\bar{x}' \in \mathcal{A}_{\text{diff}(u)} \\ \bar{x} \cup \bar{x}' \text{ is valid}}} \left[\begin{array}{l} \prod_{f \in \phi(u)} \exp(\alpha_f f(\bar{x} \cup \bar{x}')) \\ \times \prod_{c_i \text{ child of } u} \sum_{\substack{\bar{y}_i \in \mathcal{A}_{\text{diff}(T_{c_i})} \\ \bar{x} \cup \bar{x}' \cup \bar{y}_i \text{ is valid}}} \prod_{f \in \phi(T_{c_i})} \exp(\alpha_f f(\bar{x} \cup \bar{x}' \cup \bar{y}_i)) \end{array} \right]$$

As the set $\text{diff}(T_u) \setminus \text{diff}(u)$ is disjointly decomposed into $\text{diff}(T_{c_1}) \ldots \text{diff}(T_{c_k})$, the second term in the bracket can be seen as the Cartesian product of k sets. We can move the summations in front of the production,

$$= \sum_{\substack{\bar{x}' \in \mathcal{A}_{\text{diff}(u)} \\ \bar{x} \cup \bar{x}' \text{ is valid}}} \left[\begin{array}{l} \prod_{f \in \phi(u)} \exp(\alpha_f f(\bar{x} \cup \bar{x}')) \\ \times \sum_{\substack{\bar{y}_1 \in \mathcal{A}_{\text{diff}(T_{c_1})} \\ \bar{x} \cup \bar{x}' \cup \bar{y}_1 \text{ is valid}}} \cdots \sum_{\substack{\bar{y}_k \in \mathcal{A}_{\text{diff}(T_{c_k})} \\ \bar{x} \cup \bar{x}' \cup \bar{y}_k \text{ is valid}}} \prod_{c_i \text{ child of } u} \prod_{f \in \phi(T_{c_i})} \exp(\alpha_f f(\bar{x} \cup \bar{x}' \cup \bar{y}_i)) \end{array} \right]$$

For every fixed assignment $\bar{x} \cup \bar{x}'$, the product over the functions of bag u is independent of the summations. Thus, we can move the summations over the assignments $\bar{y}_i$ in front of the product,

$$= \sum_{\substack{\bar{x}' \in \mathcal{A}_{\text{diff}(u)} \\ \bar{x} \cup \bar{x}' \text{ is valid}}} \sum_{\substack{\bar{y}_1 \in \mathcal{A}_{\text{diff}(T_{c_1})} \\ \bar{x} \cup \bar{x}' \cup \bar{y}_1 \text{ is valid}}} \cdots \sum_{\substack{\bar{y}_k \in \mathcal{A}_{\text{diff}(T_{c_k})} \\ \bar{x} \cup \bar{x}' \cup \bar{y}_k \text{ is valid}}} \left[\begin{array}{l} \prod_{f \in \phi(u)} \exp(\alpha_f f(\bar{x} \cup \bar{x}')) \\ \times \prod_{c_i \text{ child of } u} \prod_{f \in \phi(T_{c_i})} \exp(\alpha_f f(\bar{x} \cup \bar{x}' \cup \bar{y}_i)) \end{array} \right]$$

Then, we combine the summations over the single partial assignments $\bar{y}_i$; moreover we replace the products with a single product ranging over the combined assignments $\bar{y}$ of $\text{diff}(T_u) \setminus \text{diff}(u)$ as $\phi(T_u) \setminus \phi(u)$ is partitioned into $\phi(T_{c_i})$, $i = 1 \ldots k$.

$$= \sum_{\substack{\bar{x}' \in \mathcal{A}_{\text{diff}(u)} \\ \bar{x} \cup \bar{x}' \cup \bar{y} \text{ is valid}}} \sum_{\substack{\bar{y} \in \mathcal{A}_{\text{diff}(T_u) \setminus \text{diff}(u)} \\ \bar{x} \cup \bar{x}' \cup \bar{y} \text{ is valid}}} \left[\prod_{f \in \phi(T_u)} \exp(\alpha_f f(\bar{x} \cup \bar{x}' \cup \bar{y})) \right]$$

The last term equals r.h.s. of Eq. (5), showing the induction step.

□

3 Correctness of sampling by stochastic traceback

Lemma S2. At edge $u \to v$, Alg. 3 choses a partial assignment $\bar{x} \in \mathcal{A}_{\text{diff}(u)}$ with probability

$$\mathbb{P}_{\text{alg}}(\bar{x} \mid x) = \frac{w_{u \to v}(\bar{x} \mid x)}{m_{u \to v}(x)}$$

where x is the partial assignment determined in the previous iterations and weight $w_{u\to v}(\bar{x} \mid x)$ is defined as

$$w_{u\to v}(\bar{x} \mid x) = \prod_{f\in\phi(u)} \exp(\alpha_f f(x\cup\bar{x})) \prod_{c \text{ child of } u} m_{c\to u}(x\cup\bar{x}) = \sum_{\substack{\bar{y}\in\mathcal{A}_{\mathrm{diff}(T_u)\setminus\mathrm{diff}(u)} \\ x\cup\bar{x}\cup\bar{y} \text{ is valid}}} \prod_{f\in\phi(T_u)} \exp(\alpha_f f(x\cup\bar{x}\cup\bar{y})). \tag{S1}$$

Proof. Let us order the partial assignments $\bar{x}_i$ in $\mathcal{A}_{\mathrm{diff}(u)}$ with i from 1 to $|\mathcal{A}_{\mathrm{diff}(u)}|$, each is associated with weight $w_{u\to v}(\bar{x}_i \mid x) = \prod_{f\in\phi(u)} \exp(\alpha_f f(x\cup\bar{x}_i)) \prod_{c \text{ child of } u} m_{c\to u}(x\cup\bar{x}_i)$. Note that the sum of all weights $\sum_{i=1}^{|\mathcal{A}_{\mathrm{diff}(u)}|} w_{u\to v}(\bar{x}_i \mid x)$ equals to $m_{u\to v}(x)$.

For a selected value t, Alg. 3 enumerates assignments $\bar{x}_i$ starting from $i = 1$. In every iteration, it subtracts the weight $w_{u\to v}(\bar{x}_i \mid x)$. It is terminated as soon as t is negative, let this index be j, and assigns $\bar{x}_j$ to $\mathrm{diff}(u)$. In other words, $\bar{x}_j$ is assigned to $\mathrm{diff}(u)$ if

$$\sum_{i=0}^{j} w_{u\to v}(\bar{x}_i \mid x) \le t < \sum_{i=0}^{j+1} w_{u\to v}(\bar{x}_i \mid x)$$

with $w_{u\to v}(\bar{x}_0 \mid x)$ is defined as 0 for $i = 0$. Since t is randomly and uniformly selected from 0 to $m_{u\to v}(x)$, the probability that Alg. 3 chooses $\bar{x}$ is

$$\mathbb{P}_{\mathrm{alg}}(\bar{x} \mid x) = \frac{w_{u\to v}(\bar{x} \mid x)}{m_{u\to v}(x)}.$$

Finally, the weight $w_{u\to v}(\bar{x} \mid x) = \prod_{f\in\phi(u)} \exp(\alpha_f f(x\cup\bar{x})) \prod_{c \text{ child of } u} m_{c\to u}(x\cup\bar{x})$ can be seen as a special case of Eq. 6 by replacing the partial assignment set $\mathcal{A}_{\mathrm{diff}(u)}$ with a singleton $\{\bar{x}\}$. Applying Proposition 2 gives

$$w_{u\to v}(\bar{x} \mid x) = \sum_{\substack{\bar{y}\in\mathcal{A}_{\mathrm{diff}(T_u)\setminus\mathrm{diff}(u)} \\ x\cup\bar{x}\cup\bar{y} \text{ is valid}}} \prod_{f\in\phi(T_u)} \exp(\alpha_f f(x\cup\bar{x}\cup\bar{y})).$$

□

Proof of Proposition 3. Let us assume that the non-root nodes of the cluster tree are labeled in preorder as $u_1, u_2, \ldots, u_\ell$. Alg. 3 assigns values in the order of $\mathrm{diff}(u_1), \ldots, \mathrm{diff}(u_\ell)$; it finally generates a total assignment since $\cup_{i=1}^{\ell} \mathrm{diff}(u_i) = \mathcal{X}$.

Let $x^* = \bar{x}_1^* \cup \cdots \cup \bar{x}_\ell^*$ be a total assignment where $\bar{x}_i^*$ is a partial assignment of $\mathcal{A}_{\mathrm{diff}(u_i)}$ for i from 1 to ℓ. The probability of x^* can be written as product of conditional probabilities

$$\mathbb{P}(x^*) = \mathbb{P}(\bar{x}_1^*)\,\mathbb{P}(\bar{x}_2^* \mid \bar{x}_1^*) \cdots \mathbb{P}(\bar{x}_\ell^* \mid \cup_{k=1}^{\ell-1}\bar{x}_k^*) = \cup_{i=1}^{\ell}\,\mathbb{P}(\bar{x}_i^* \mid \cup_{k=1}^{i-1}\bar{x}_k^*)$$

with the conditional Boltzmann probability

$$\mathbb{P}(\bar{x}_i^* \mid \cup_{k=1}^{i-1}\bar{x}_k^*) = \frac{\sum_{\substack{\bar{x}\in\mathcal{A}_{\mathrm{diff}(u_{i+1})\cup\ldots\cup\mathrm{diff}(u_\ell)} \\ \bar{x} \text{ is valid}}} \prod_{f\in\phi_{\mathrm{all}}} \exp(\alpha_f f(\cup_{k=1}^{i-1}\bar{x}_k^* \cup \bar{x}_i^* \cup \bar{x}))}{\sum_{\substack{\bar{x}'\in\mathcal{A}_{\mathrm{diff}(u_i)} \\ \bar{x}' \text{ is valid}}} \sum_{\substack{\bar{x}\in\mathcal{A}_{\mathrm{diff}(u_{i+1})\cup\ldots\cup\mathrm{diff}(u_\ell)} \\ \bar{x} \text{ is valid}}} \prod_{f\in\phi_{\mathrm{all}}} \exp(\alpha_f f(\cup_{k=1}^{i-1}\bar{x}_k^* \cup \bar{x}' \cup \bar{x}))} \tag{S2}$$

where ϕ_{all} is the set of all network functions in the feature network. Note that for $i = 1$, the union $\cup_{k=1}^{0} x_k^*$ is empty.

At the edge $u_i \to v$, where the variables $\mathrm{diff}(u_1), \ldots, \mathrm{diff}(u_{i-1})$ have been assigned by $\bar{x}_1^*, \ldots, \bar{x}_{i-1}^*$, the probability that Alg. 3 chooses $\bar{x}_i^*$ for $\mathrm{diff}(u_i)$ is given by Lemma S2 as

$$\mathbb{P}_{\mathrm{alg}}(\bar{x}_i^* \mid \cup_{k=1}^{i-1}\bar{x}_k^*) = \frac{w_{u_i\to v}(\bar{x}_i^* \mid \cup_{k=1}^{i-1}\bar{x}_k^*)}{m_{u_i\to v}(\cup_{k=1}^{i-1}\bar{x}_k^*)}. \tag{S3}$$

Our goal is to show that two conditional probabilities $\mathbb{P}(\bar{x}_i^* \mid \cup_{k=1}^{i-1}\bar{x}_k^*)$ of Eq. S2 and $\mathbb{P}_{\mathrm{alg}}(\bar{x}_i^* \mid \cup_{k=1}^{i-1}\bar{x}_k^*)$ of Eq. S3 are actually the same.

We rewrite $\mathbb{P}_{\mathrm{alg}}$ of Eq. S3 using Eq. S1 and the definition of conditional partition function as in Eq. 5

$$\mathbb{P}_{\text{alg}}(\bar{x}_i^* \mid \cup_{k=1}^{i-1}\bar{x}_k^*)$$

$$= \frac{\sum_{\substack{\bar{y}\in\mathcal{A}_{\text{diff}(T_{u_i})\setminus\text{diff}(u_i)} \\ x\cup\bar{x}_i^*\cup\bar{y} \text{ is valid}}} \prod_{f\in\phi(T_{u_i})} \exp(\alpha_f f(\cup_{k=1}^{i-1}\bar{x}_k^* \cup \bar{x}_i^* \cup \bar{y}))}{\sum_{\substack{\bar{x}'\in\mathcal{A}_{\text{diff}(u_i)} \\ \bar{x}' \text{ is valid}}} \sum_{\substack{\bar{y}\in\mathcal{A}_{\text{diff}(T_{u_i})\setminus\text{diff}(u_i)} \\ x\cup\bar{x}'\cup\bar{y} \text{ is valid}}} \prod_{f\in\phi(T_{u_i})} \exp(\alpha_f f(\cup_{k=1}^{i-1}\bar{x}_k^* \cup \bar{x}' \cup \bar{y}))}$$

Comparing with $\mathbb{P}(\bar{x}_i^* \mid \cup_{k=1}^{i-1}\bar{x}_k^*)$ of Eq. S2, only a subset of network functions $\phi(T_u)$ are taken into account. We are going to bring the rest into the expression. First, let us consider the functions assigned to the nodes that Alg. 3 has visited before edge $u_i \to v$, denoted by $\phi_{\text{visited}} := \cup_{k=1}^{i-1}\phi(u_k)$. Multiplying nominator and denominator by partial partition function $\prod_{f\in\phi_{\text{visited}}} \exp(\alpha_f f(\cup_{k=1}^{i-1}\bar{x}_k^*))$ restricted to ϕ_{visited} and the partial assignment $\cup_{k=1}^{i-1}\bar{x}_k^*$ gives

$$= \frac{\left(\sum_{\substack{\bar{y}\in\mathcal{A}_{\text{diff}(T_{u_i})\setminus\text{diff}(u_i)} \\ x\cup\bar{x}_i^*\cup\bar{y} \text{ is valid}}} \prod_{f\in\phi(T_{u_i})} \exp(\alpha_f f(\cup_{k=1}^{i-1}\bar{x}_k^* \cup \bar{x}_i^* \cup \bar{y}))\right)\left(\prod_{f\in\phi_{\text{visited}}} \exp(\alpha_f f(\cup_{k=1}^{i-1}\bar{x}_k^*))\right)}{\left(\sum_{\substack{\bar{x}'\in\mathcal{A}_{\text{diff}(u_i)} \\ \bar{x}' \text{ is valid}}} \sum_{\substack{\bar{y}\in\mathcal{A}_{\text{diff}(T_{u_i})\setminus\text{diff}(u_i)} \\ x\cup\bar{x}'\cup\bar{y} \text{ is valid}}} \prod_{f\in\phi(T_{u_i})} \exp(\alpha_f f(\cup_{k=1}^{i-1}\bar{x}_k^* \cup \bar{x}' \cup \bar{y}))\right)\left(\prod_{f\in\phi_{\text{visited}}} \exp(\alpha_f f(\cup_{k=1}^{i-1}\bar{x}_k^*))\right)}$$

As $\text{diff}(T_{u_i})$ is disjoint to the dependency of any function of ϕ_{visited}, we can further group the products

$$= \frac{\sum_{\substack{\bar{y}\in\mathcal{A}_{\text{diff}(T_{u_i})\setminus\text{diff}(u_i)} \\ x\cup\bar{x}_i^*\cup\bar{y} \text{ is valid}}} \prod_{f\in\phi_{\text{visited}}\cup\phi(T_{u_i})} \exp(\alpha_f f(\cup_{k=1}^{i-1}\bar{x}_k^* \cup \bar{x}_i^* \cup \bar{y}))}{\sum_{\substack{\bar{x}'\in\mathcal{A}_{\text{diff}(u_i)} \\ \bar{x}' \text{ is valid}}} \sum_{\substack{\bar{y}\in\mathcal{A}_{\text{diff}(T_{u_i})\setminus\text{diff}(u_i)} \\ x\cup\bar{x}'\cup\bar{y} \text{ is valid}}} \prod_{f\in\phi_{\text{visited}}\cup\phi(T_{u_i})} \exp(\alpha_f f(\cup_{k=1}^{i-1}\bar{x}_k^* \cup \bar{x}' \cup \bar{y}))}$$

We consider now the rest of the network functions, denoted by ϕ_{rest}. These functions are assigned to the cluster nodes, denoted by $\mathcal{U}_{\text{rest}}$, that have not been visited before the edge $u_i \to v$ or are not in the subtree of u_i. Note that $T_{u_i} \cup \mathcal{U}_{\text{rest}} = \{u_i, \ldots, u_\ell\}$. The partial partition function restricted to ϕ_{rest} for fixed partial assignment $\cup_{k=1}^{i-1}\bar{x}_k^*$ is

$$\sum_{\substack{\bar{y}'\in\mathcal{A}_{\cup_{u\in\mathcal{U}_{\text{rest}}}\text{diff}(u)} \\ \bar{y}' \text{ is valid}}} \prod_{f\in\phi_{\text{rest}}} \exp(\alpha_f f(\cup_{k=1}^{i-1}\bar{x}_k^* \cup \bar{y}')).$$

Multiplying again the partial partition function on nominator and denominator gives

$$= \frac{\left(\sum_{\substack{\bar{y}\in\mathcal{A}_{\text{diff}(T_{u_i})\setminus\text{diff}(u_i)} \\ x\cup\bar{x}_i^*\cup\bar{y} \text{ is valid}}} \prod_{f\in\phi_{\text{visited}}\cup\phi(T_{u_i})} \exp(\alpha_f f(\cup_{k=1}^{i-1}\bar{x}_k^* \cup \bar{x}_i^* \cup \bar{y}))\right)\left(\sum_{\substack{\bar{y}'\in\mathcal{A}_{\cup_{u\in\mathcal{U}_{\text{rest}}}\text{diff}(u)} \\ \bar{y}' \text{ is valid}}} \prod_{f\in\phi_{\text{rest}}} \exp(\alpha_f f(\cup_{k=1}^{i-1}\bar{x}_k^* \cup \bar{y}'))\right)}{\left(\sum_{\substack{\bar{x}'\in\mathcal{A}_{\text{diff}(u_i)} \\ \bar{x}' \text{ is valid}}} \sum_{\substack{\bar{y}\in\mathcal{A}_{\text{diff}(T_{u_i})\setminus\text{diff}(u_i)} \\ x\cup\bar{x}'\cup\bar{y} \text{ is valid}}} \prod_{f\in\phi_{\text{visited}}\cup\phi(T_{u_i})} \exp(\alpha_f f(\cup_{k=1}^{i-1}\bar{x}_k^* \cup \bar{x}' \cup \bar{y}))\right)\left(\sum_{\substack{\bar{y}'\in\mathcal{A}_{\cup_{u\in\mathcal{U}_{\text{rest}}}\text{diff}(u)} \\ \bar{y}' \text{ is valid}}} \prod_{f\in\phi_{\text{rest}}} \exp(\alpha_f f(\cup_{k=1}^{i-1}\bar{x}_k^* \cup \bar{y}'))\right)}$$

Because of the disjointness between $\text{diff}(T_{u_i}) \setminus \text{diff}(u_i)$ and $\cup_{u\in\mathcal{U}_{\text{rest}}}\text{diff}(u)$, we can group the products and combine the partial assignments into one $\bar{x} = \bar{y} \cup \bar{y}'$ as

$$= \frac{\sum_{\substack{\bar{x}\in\mathcal{A}_{\text{diff}(u_{i+1})\cup\ldots\cup\text{diff}(u_\ell)} \\ \bar{x} \text{ is valid}}} \prod_{f\in\phi_{\text{all}}} \exp(\alpha_f f(\cup_{k=1}^{i-1}\bar{x}_k^* \cup \bar{x}_i^* \cup \bar{x}))}{\sum_{\substack{\bar{x}'\in\mathcal{A}_{\text{diff}(u_i)} \\ \bar{x}' \text{ is valid}}} \sum_{\substack{\bar{x}\in\mathcal{A}_{\text{diff}(u_{i+1})\cup\ldots\cup\text{diff}(u_\ell)} \\ \bar{x} \text{ is valid}}} \prod_{f\in\phi_{\text{all}}} \exp(\alpha_f f(\cup_{k=1}^{i-1}\bar{x}_k^* \cup \bar{x}' \cup \bar{x}))}.$$

Thus, $\mathbb{P}_{\text{alg}}(\bar{x}_i^* \mid \cup_{k=1}^{i-1}\bar{x}_k^*) = \mathbb{P}(\bar{x}_i^* \mid \cup_{k=1}^{i-1}\bar{x}_k^*)$. □
